# The Serine Protease Homolog, Scarface, Is Sensitive to Nutrient Availability and Modulates the Development of the *Drosophila* Blood–Brain Barrier

**DOI:** 10.1523/JNEUROSCI.0452-20.2021

**Published:** 2021-07-28

**Authors:** Esteban G. Contreras, Álvaro Glavic, Andrea H. Brand, Jimena A. Sierralta

**Affiliations:** ^1^Biomedical Neuroscience Institute and Department of Neuroscience, Faculty of Medicine, Universidad de Chile, Santiago 8380453, Chile; ^2^Fund for Research Centers in Prioritary Areas Center for Genome Regulation, Faculty of Science, Universidad de Chile, Santiago 7800024, Chile; ^3^The Gurdon Institute and Department of Physiology, Development and Neuroscience, University of Cambridge, Cambridge CB2 1QN, England

**Keywords:** blood–brain barrier, *Drosophila melanogaster*, glial cells, nutrient restriction, Scarface, serine protease homolog

## Abstract

The adaptable transcriptional response to changes in food availability not only ensures animal survival but also lets embryonic development progress. Interestingly, the CNS is preferentially protected from periods of malnutrition, a phenomenon known as “brain sparing.” However, the mechanisms that mediate this response remain poorly understood. To get a better understanding of this, we used *Drosophila melanogaster* as a model, analyzing the transcriptional response of neural stem cells (neuroblasts) and glia of the blood–brain barrier (BBB) from larvae of both sexes during nutrient restriction using targeted DamID. We found differentially expressed genes in both neuroblasts and glia of the BBB, although the effect of nutrient deficiency was primarily observed in the BBB. We characterized the function of a nutritional sensitive gene expressed in the BBB, the serine protease homolog, *scarface* (*scaf*). Scaf is expressed in subperineurial glia in the BBB in response to nutrition. Tissue-specific knockdown of *scaf* increases subperineurial glia endoreplication and proliferation of perineurial glia in the blood–brain barrier. Furthermore, neuroblast proliferation is diminished on *scaf* knockdown in subperineurial glia. Interestingly, reexpression of Scaf in subperineurial glia is able to enhance neuroblast proliferation and brain growth of animals in starvation. Finally, we show that loss of *scaf* in the blood–brain barrier increases sensitivity to drugs in adulthood, suggesting a physiological impairment. We propose that Scaf integrates the nutrient status to modulate the balance between neurogenesis and growth of the BBB, preserving the proper equilibrium between the size of the barrier and the brain.

**SIGNIFICANCE STATEMENT** The *Drosophila* BBB separates the CNS from the open circulatory system. The BBB glia are not only acting as a physical segregation of tissues but participate in the regulation of the metabolism and neurogenesis during development. Here we analyze the transcriptional response of the BBB glia to nutrient deprivation during larval development, a condition in which protective mechanisms are switched on in the brain. Our findings show that the gene *scarface* reduces growth in the BBB while promoting the proliferation of neural stem, assuring the balanced growth of the larval brain. Thus, Scarface would link animal nutrition with brain development, coordinating neurogenesis with the growth of the BBB.

## Introduction

The formation of the nervous system is a tightly regulated process that is controlled by complex mechanisms that mitigate external perturbations, such as temperature changes and food availability. For example, the developing mammalian brain is protected against intrauterine growth restriction by a phenomenon known as brain sparing in which the animal adapts to maintain oxygen and nutrient levels in the brain (Cohen et al., 2015). Brain development and function requires a microenvironment that is established and maintained by the blood–brain barrier (BBB), a selective barrier that separates the nervous system from the circulating blood. Therefore, the BBB could act as a metabolic sensor to protect the nervous system against a decrease in nutrient availability.

The mammalian BBB is established by endothelial cells forming tight junctions that prevent the paracellular diffusion of macromolecules and ions. Pericytes and astrocyte projections modulate the function of the BBB (Zhao et al., 2015; Gürsoy-özdemir and Tas, 2017; Haddad-Tóvolli et al., 2017), creating a selective barrier and establishing an homeostatic milieu independent from the rest of the body. The insect BBB covers the entire nervous system to isolate it from the hemolymph (the insect blood; Carlson et al., 2000) and performs similar functions in the mammalian BBB. In insects, the BBB is formed by two layers of glia, perineurial (PG) and subperineurial (SPG; Awasaki et al., 2008; Stork et al., 2008; Hindle and Bainton, 2014; Schirmeier and Klämbt, 2015; O'Brown et al., 2018; Yildirim et al., 2019). The PG form the outer layer of the BBB, which regulates the transport of nutrients and secretes components of the extracellular matrix (the neural lamella; DeSalvo et al., 2014; Volkenhoff et al., 2015; Kanai et al., 2018). The inner layer, the SPG, forms septate junctions that block the passive movement of solutes across the BBB (Baumgartner et al., 1996; Carlson et al., 2000; Schwabe et al., 2005; Stork et al., 2008), controlling nutrient entry (Galagovsky et al., 2018) and the excretion of xenobiotic molecules (Tapadia and Lakhotia, 2005; Mayer et al., 2009; Hindle et al., 2017).

During development, the BBB not only acts as a barrier but also influences the rate of neurogenesis by secreting growth factors that stimulate neurogenesis (Zhu et al., 2008; Chell and Brand, 2010; Sousa-Nunes et al., 2011; Spéder and Brand, 2014; Kanai et al., 2018). BBB glia mediate the reactivation of neural stem cells [neuroblasts (NB)] from a period of quiescence in response to nutrition (Britton and Edgar, 1998; Chell and Brand, 2010; Sousa-Nunes et al., 2011). Periods of starvation during early larval development block neurogenesis by arresting NBs in a quiescent state. However, by third instar larval stages, NB are insensitive to undernourishment and continue proliferating even after complete starvation, a phenomenon that resembles mammalian brain sparing (Cheng et al., 2011; Lanet et al., 2013; Lanet and Maurange, 2014; Contreras et al., 2018). This protocol of complete starvation is very effective in reducing the levels of carbohydrates, amino acids, and proteins in the larval hemolymph (Cheng et al., 2011; Handke et al., 2013; Yamada et al., 2018). Considering this, it is plausible that the initial response of the CNS to the decrease in systemic nutrients is triggered by the BBB glia. Therefore, understanding how BBB gene expression is modulated by nutrition may yield insights into the adaptive mechanisms that govern brain sparing.

Here, we performed cell-type-specific transcriptional analysis of the BBB glia under fed and nutrient restriction (NR) conditions. Among the differentially expressed genes in the SPG, we found *scarface* (*scaf*), a member of the serine protease homolog (SPH) family. We show that *scaf* expression is sensitive to nutrition during larval development. *scaf* is expressed by the SPG and is required for controlling SPG growth and PG proliferation. Moreover, Scaf is necessary for the proper rate of neurogenesis, and its overexpression enhances NB proliferation and brain growth in animals subjected to NR. Finally, we show that knocking down *scaf* in SPG affects the resistance of flies to drugs, suggesting that the function of the BBB is impaired when *scaf* is lost.

## Material and Methods

### 

#### 

##### Fly stocks and husbandry

*Drosophila melanogaster* stocks were cultured in fly food medium at 25°C. Our fly food contains the following ingredients per liter of medium: 100 g yeast, 80 g glucose, 50 g wheat flower, 11 g agar, 6 ml propionic acid, and 12 ml 20% Nipagin (methylparaben). All RNAi experiments were performed at 29°C.

For Targeted DNA adenine methyltransferase identification (DamID; TaDa) analysis, we used *tub-GAL80^ts^, UAS-LT3-NDam-RpII215* (*NDam-PolII*), and *tub-GAL80^ts^, UAS-LT3-NDam* (Southall et al., 2013) crossed to the drivers *wor-GAL4* (Albertson et al., 2004), *mdr65*/*R54C07-GAL4* (Jenett et al., 2012; Spéder and Brand, 2014), or *sema5c/R71C08-GAL4* (Jenett et al., 2012). For functional experiments we used *w^1118^* as experimental control, *UAS-shScaf^RNAi^* (catalog #330286, Vienna Drosophila Resource Center), *UAS-lhScaf^RNAi^* (catalog #13249, Vienna Drosophila Resource Center), *UAS-DlgA::EGFP* (Koh et al., 1999), *UAS-GFP.nls* (catalog #107-870, Kyoto Stock Center), *UAS-mCD8-GFP*, *UAS-mCD8-RFP*, *UAS-lam:GFP* (catalog #7378, Bloomington Drosophila Stock Center), and *UAS-Scaf::GFP* (Rousset et al., 2010). For fluorescent reporters and fusion proteins we used the following stocks: *scaf^PBss^* (Scaf::GFP; Bonin and Mann, 2004), *10xSTAT92E-GFP* (Bach et al., 2007), *TRE-RFP* (Chatterjee and Bohmann, 2012), *LanA::GFP* (catalog #318155, Vienna Drosophila Resource Center), *Lac::GFP^G00044^* (Morin et al., 2001), and *mdr65-mtdTomato* (Benmimoun et al., 2020). We used the glial drivers *R54C07-GAL4* (*mdr65-GAL4*; catalog #50472, Bloomoington Drosophila Stock Center) and *moody-GAL4* (Schwabe et al., 2005) for knockdown and overexpression experiments. We used the mutant alleles *scaf^27^* (Rousset et al., 2010) and *scaf^MI09409^* (catalog #53101, Bloomoington Drosophila Stock Center).

##### Nutrient restriction protocol

Nutrient restriction (NR) experiments were performed as previously described (Contreras et al., 2018). Briefly, 68–72 h after larval hatching (ALH), larvae were transferred to a tube with fly food (Fed) or 1% Agarose in × PBS (NR). Tubes were left at 25°C for 24 h or until pupariation. For adult dissection, pupae were transferred to food tubes, and adult fly brains were dissected 1–3 d after eclosion.

##### Sample collection for targeted DamID

Briefly, we crossed *tub-GAL80^ts^; UAS-LT3-NDam* and *tub-GAL80^ts^; UAS-LT3-NDam-RpII215* to the respective drivers. We collected embryos for 3 h and let them develop for 8 d at 18°C. Third instar larvae were transferred to 29°C for 1 d to induce the expression of the TaDa construct in either Fed or NR (1% Agarose, 1 × PBS) conditions. Fifty to 70 brains of each condition were dissected to extract genomic DNA. Two to five replicates were processed.

##### DamID sequencing, processing and data analysis

The isolated genomic DNA from each cross was digested and amplified to generate libraries for next-generation sequencing as described (Marshall et al., 2016). Libraries were sequenced in an Illumina HiSeq 1500 with at least 8 million reads per library.

TaDa reads were aligned to *Drosophila melanogaster* genome release dm6 and normalized using the damidseq_pipeline (Marshall and Brand, 2015). Genome browser views of bedgraph files were displayed as Log_2_(DamPolII/Dam) using Integrative Genomics Viewer software (Robinson et al., 2011). RNA polymerase II (PolII)-bound genes were called using the polii.gene.call Rscript (Marshall and Brand, 2015; Marshall et al., 2016) using *Drosophila* genome Annotation Release dm6.06. Genes with a false discovery Rate (FDR) lower than 0.01 were considered as bound by PolII. All genes from two datasets were compared using polii.correlation.plot R script (Marshall and Brand, 2015). Differentially bound genes have a difference between Log_2_(Dam-PolII/Dam) values of 0.3, an FDR < 0.01 in at least one dataset, and a ratio of PolII binding bigger than 2. Student's test was used to analyze *p* values between Fed and NR replicates.

Gene ontology analysis of differentially expressed genes was performed using the Database for Annotation, Visualization, and Integrated Discovery Bioinformatics Resources 6.8 (Huang et al., 2009a,b). Association networks were constructed using String (Szklarczyk et al., 2019). Plots and heatmaps using custom R scripts are available on request. PolII average Log_2_(Dam-PolII/Dam) from each replicate were correlated using Pearson's method and displayed as a heatmap using R gplots library. Volcano plots were generated using the R ggplot2 library. Student's test *p* values were used to compare every replicate from two conditions (Fed and NR) and the difference of Log_2_(Dam-PolII/Dam) for each gene in the conditions. Heatmaps were created using the R pheatmap library, and for clustering genes the hclust complete method was used.

##### Immunostaining

Third instar larval brains were fixed in 4% formaldehyde for 20 min and stained as previously described (Wu and Luo, 2006). The following primary antibodies were used: rat anti-N-cadherin (CadN; 1:20; catalog #DN-Ex #8, Developmental Studies Hybridoma Bank), guinea pig anti-Deadpan (Dpn; 1:5000; Andrea Brand Lab), rat anti-Elav (1:20; catalog #7E8A10, Developmental Studies Hybridoma Bank), mouse anti-Fas2 (1:20; catalog #1D4, Developmental Studies Hybridoma Bank), rabbit anti-phospho-Histone H3 (1:400; catalog #06–570 Merck), rat anti-phospho-Histone H3 (1:500; catalog #HTA28, Abcam), mouse anti-Repo (1:20; catalog #8D12, Developmental Studies Hybridoma Bank), and rabbit anti-Scaf N°2 (1:200; Rousset et al., 2010). DNA was stained using TO-PRO−3 (1:400; catalog #T3605, Invitrogen) or 0.2 µg/ml DAPI. FITC, Cy3, or Cy5 conjugated secondary antibodies were used at a final concentration of 1:200 (Jackson ImmunoResearch).

For BBB permeability assays, larvae were opened at the tail and inverted, exposing the brain. Larvae were incubated with 20 µg/ml Rhodamine-dextran (10,000 MW; #D1863, Invitrogen,) in Schneider's insect medium for 30 min at room temperature. Brains were washed in 1 × PBS and fixed in 4% formaldehyde in 1 × PBS for 20 min. Samples were washed in PBT (0.3% Triton X-100, 1 × PBS). Brains were dissected and mounted in VECTASHIELD (Vector). For adult animals, males were anesthetized with CO_2_, and ∼60 nL of 12.5 µg/ml Rhodamine-dextran 10,000 MW was injected in the abdomen using a Nanoject II Auto-Nanoliter Injector (Drummond Scientific). Flies were left to recover 2 h at room temperature, and brains were dissected and fixed.

##### Imaging

Images were acquired using a Leica SP8 or an Olympus FluoView FV1000 scanning confocal microscope. Images of wings, pupae, and adult flies were taken using a Nikon stereomicroscope with a Canon Rebel 2 Ti camera. Images, diagrams, and figures were assembled using Fiji, Adobe Photoshop CC, and Adobe Illustrator CC.

##### Behavioral assays

Larval locomotion was performed by placing 10–12 larvae on a 1% agarose plate and recording a video for 1–2 min. Speed was calculating using the ImageJ plugin wrMTrck (Brooks et al., 2016). Adult climbing was performed using a six-tube countercurrent apparatus (Inagaki et al., 2010). Briefly, groups of 15–30 female flies were placed in the first tube of the apparatus and allowed to climb for 30 s. Flies that climbed were transferred to the next tube, and the process was repeated until the last tube was occupied. Then the number of flies in each tube was counted. The climbing index was calculated with the following equation: Ci=(N2+2N3+3N4+4N5+5N6)/5(N1+N2+N3+N4+N5+N6), in which N_1_-N_6_ corresponds to the number of flies in each of the six tubes. Eight different groups of flies were tested for each genotype. For fly activity and sleep assay, we used Drosophila Activity Monitors (DAM2, TriKinetics) and loaded adult flies following a previously described protocol (Chiu et al., 2010). Activity monitors were placed in an incubator with a 12:12 h light:dark cycle at 25°C for 6 d; only the last 4 d were used for data collection. The data were analyzed using ShinyR-DAM (Cichewicz and Hirsh, 2018). Sleep was defined as inactivity in a period of 5 min.

For ethanol sedation sensitivity assay, we used groups of 8–10 male flies, placed in an empty vial with a cellulose acetate plug. One ml of ethanol was added to the plug, and the fraction of immobile flies was counted every minute until all flies were sedated (Sandhu et al., 2015). The time at which half the flies were sedated, or sedation time 50 (ST50), was estimated by fitting a sigmoidal curve. For malathion resistance assay, we placed groups of 20 female flies in tubes containing 0.5 g of instant dry food (Carolina Biological Supply) and 2 ml 0.01% malathion in dH_2_O. Lethality was counted at 12, 24, and 36 h after malathion exposure.

##### Quantifications and data analysis

Pupal volume was estimated as previously described (Layalle et al., 2008), using the ellipsoid volume formula 4/3π(L/2)(d/2)^2^ (L, length; d, diameter). Time of pupariation analysis was performed as we previously described (González-Itier et al., 2018). Larval and adult brain size was estimated using Imaris 7 (Bitplane) as described (Contreras et al., 2018). Neuropil size was estimated using the area of CadN staining in a maximal intensity Z-projection. The SPG nuclear area was measured by generating a Z-stack maximum intensity projection and calculating the area of each SPG nucleus using Fiji (Li et al., 2017). The number of SPG nuclei was counted in a three-dimensional (3D) reconstruction using the Surpass feature of Imaris 7 (Bitplane). The number of PG was counted using *mdr65-GAL4, UAS-DlgA::GFP* or *moody-GAL4*, *UAS-GFP.nls* to mark SPG nuclei. Repo-positive/GFP-negative nuclei at the anterior surface of larval brain lobes were counted as PG. Scaf intensity was quantified by selecting the BBB optical slice, marking a region of interest of the brain lobe and calculating the mean intensity of the Scaf channel using Fiji. For analyzing the size of the wings, adult flies, 2–3 d after eclosion, were fixed in 95% ethanol for 24 h. Wings were dissected and mounted in a 1:1 lactic acid:ethanol solution. Fiji was used to measure the area of each wing.

All diagrams and figures were assembled using Fiji, Adobe Photoshop CC, and Adobe Illustrator CC. Graphs and statistical analysis were conducted using GraphPad Prism 8. Descriptions of each statistical test used are provided in the figure legends, and *p* values <0.05, <0.01, <0.001, and <0.0001 are shown in plots indicated by asterisks, whereas ns means nonsignificant (*p* > 0.05).

##### Data availability

DamID-Seq data were deposited in the Gene Expression Omnibus under the accession number GSE145055.

## Results

### A *Drosophila* brain-sparing model for understanding the response to nutrient restriction

**Figure 1.**
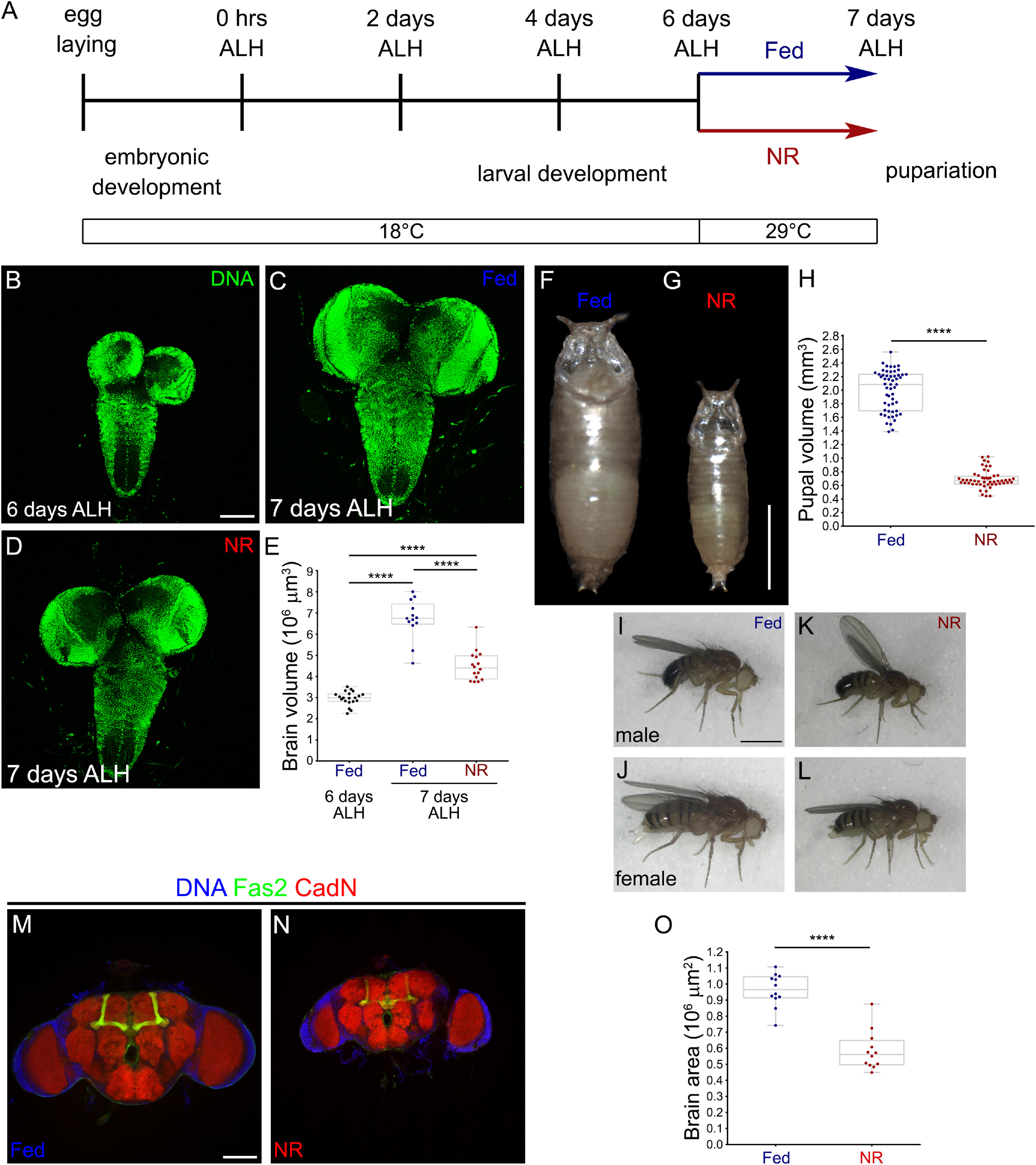
Brain sparing as a model for understanding the adaptation to nutrient restriction. ***A***, Scheme showing our NR protocol for TaDa experiments. ***B–D***, Larval brains stained for DNA (green) at (***B***) 6 d ALH, (***C***) 7 d ALH in Fed conditions, and (***D***) 7 d ALH in NR conditions. Scale bar, 100 µm. ***E***, Quantification of brain volume. *n* = 20, 13 and 16 brains. One-way ANOVA and Tukey's multiple-comparisons test were performed. ***F–G***, Comparison of pupal size on (***F***) Fed or (***G***) NR. Scale bar, 1 mm. ***H***, Graph showing the pupal volume in Fed and NR animals. *n* = 55 and 52 pupae, respectively. Mann–Whitney test was performed. ***I–L***, Representative (***I***, ***K***) male and (***J***, ***L***) female adult flies in (***I***, ***J***) Fed or (***K***, ***L***) NR conditions. ***M***, ***N***, Immunostaining against Fas2 (green), CadN (red), and DNA (blue) of adult brains of animals in (***M***) Fed or (***N***) NR conditions during larval development. Scale bar, 100 µm. ***O***, Plot showing the area of adult brains after Fed or NR protocol. *n* = 12 adult brains for each conditions. Unpaired Student's *t* test was used, ****p < 0.0001.

To understand the transcriptional response of the *Drosophila* BBB to NR, we established the brain-sparing model (Cheng et al., 2011; Contreras et al., 2018) under the temperature conditions required for performing PolII targeted TaDa (Southall et al., 2013). Thus, we allowed development at 18°C until 6 d ALH and switched to Fed or NR conditions at 29°C for 1 d (Fig. 1*A*).

Following this protocol, the consequences of NR during larval development could be observed as a reduction in the size of the animal in both pupal (Fig. 1*F–H*) and adult (Fig. 1*I–L*) stages. The average size of the pupa after NR (pupal size reflects the maximum size that a larva reached before pupariation) corresponded to 34.51% of Fed animals (Fig. 1*H*). We found that under this condition, larval brains continued growing on NR but not at the same rate as their Fed counterparts (Fig. 1*B–D*). For the adult brain, we observed a significant reduction in the brain size of animals that were subjected to NR during larval development (Fig. 1*M*,*N*). The larval and adult brains of NR animals reached 67.08% and 60.62% of the size of the Fed brains, respectively (Fig. 1*E*,*O*). Therefore, we were able to replicate the brain sparing effect under the conditions required to perform TaDa as the effect of NR over the growth of the whole animal was greater compared with the brain (body reduction to almost 35% compared with brain reduction to a 67% of the Fed animal), reflecting the preferential growth of the nervous system during starvation (Cheng et al., 2011).

### Transcriptional profiling of neuroblasts and blood–brain barrier glial cells under nutrient restriction

**Figure 2.**
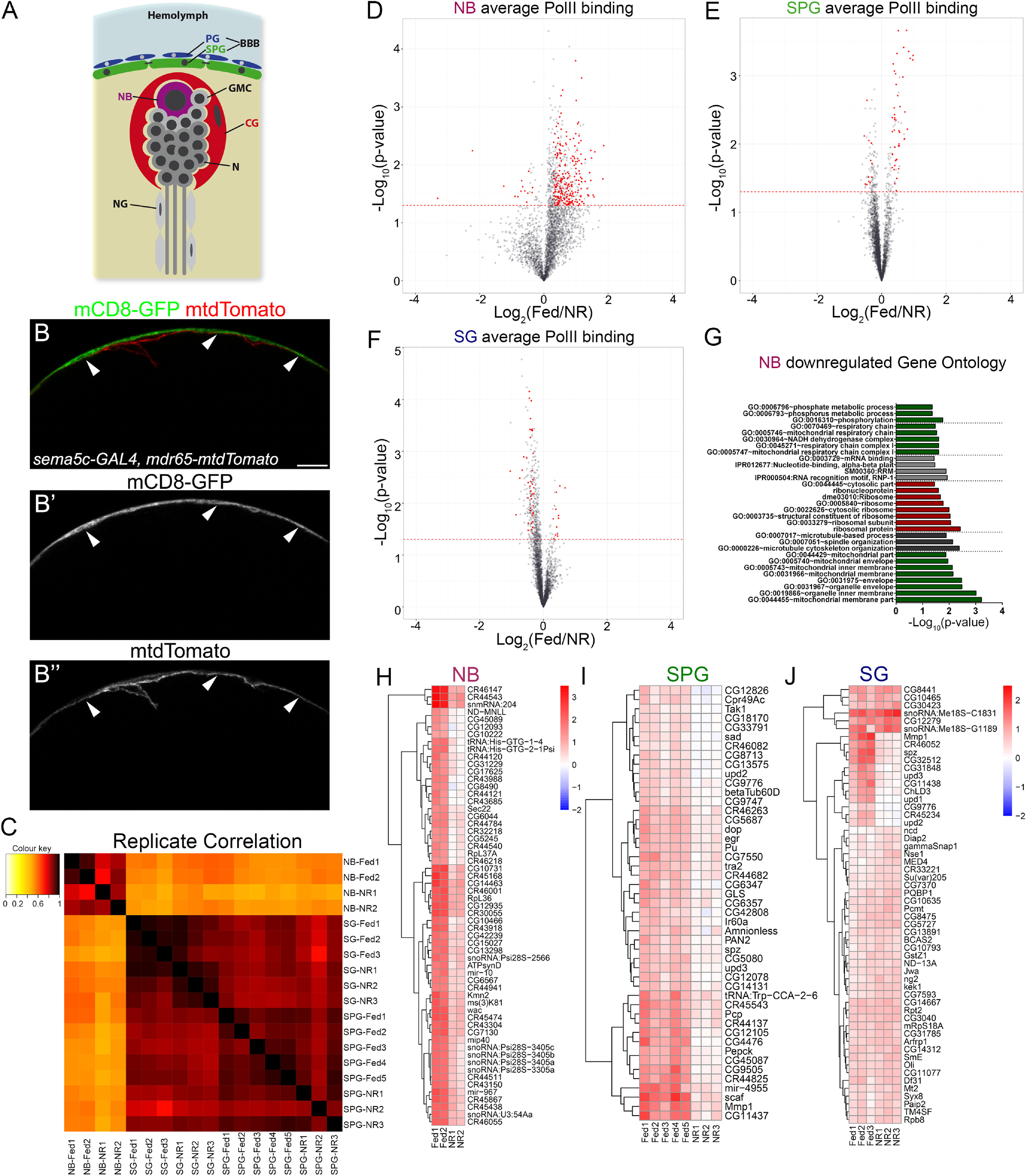
Targeted DamID in neuroblasts and glial cells of the blood–brain barrier after nutrient restriction. ***A***, Schematic representation of the different cell types in the larval brain. CG, Cortex glia; GMC, ganglion mother cells; N, neuron; NG, neuropil glia (Extended Data Figure 2-1, schematic representation). ***B***, ***B*'**, ***B*''**, Confocal images of larval brain expressing mCD8-GFP in superficial glia (PG and SPG, *sema5c-GAL4, UAS-mCD8-GFP,* green and gray) and mtdTomato in SPG (*mdr65-mtdTomato*, red and gray). Arrowheads show the overlap of the green and red signals. Scale bar, 20 µm. PolII binding in BBB marker genes shown in Extended Data Figure 2-1. ***C***, Heatmap diagram showing correlation of all Fed and NR TaDa libraries. ***D–F***, Volcano plots showing PolII bound genes in NR and Fed conditions in (***D***) NBs, (***E***) SPG, and (***F***) surface glia (SPG+PG). Genes differentially bound by PolII between Fed and NR conditions are highlighted in red. ***G***, Graph showing the gene ontology categories enriched in genes downregulated in NBs by NR (Extended Data Figure 2-1). ***H–J***, Heatmaps showing the PolII binding of differentially expressed genes in the TaDa replicates of (***H***) NBs, (***I***) SPG, and (***J***) SG. Tables of PolII binding in all genes are shown in Extended Data Figure 2-2.

To determine the transcriptional response of neuroblasts and the BBB to NR, we profiled *in vivo,* and in a cell-type-specific manner, the binding of RNA Polymerase II (RpII215/PolII) using the TaDa technique. Briefly, TaDa uses the GAL4 system (Brand and Perrimon, 1993) to drive expression of a DNA-binding or chromatin factor fused to the *Escherichia coli* Dam methylase (Southall et al., 2013; Aughey and Southall, 2016; van den Ameele et al., 2019; Extended Data Fig. 2-1*A*,*B*). We used two GAL4 drivers to target NDam-PolII expression in the SPG (*R54C07-GAL4*, also referred to as *mdr65-GAL4*) or the PG and SPG together (SG, *R71C08-GAL4*, *sema5c-GAL4*; Fig. 2*B–B*'' for driver expression; Extended Data Fig. 2-1*D–G* for PolII binding in BBB genes), and *wor-GAL4* for profiling NDam-PolII binding in all NBs (Extended Data Fig. 2-1, Fig. 2*A*). We performed TaDa under two nutritional conditions, Fed and NR, according to our established protocol (Fig. 1*A*).

10.1523/JNEUROSCI.0452-20.2021.f2-1 Figure 2-1 ***A***, Drivers used for TaDa in different cell populations. ***B***, Scheme of the TaDa mechanism showing the binding of the NDam-PolII fusion protein to the DNA and the methylation in GATC sequence. ***C***, Meta-analysis of the NDam-PolII binding using the driver *wor-GAL4* [neural stem cells (NSC), neuroblast]. Graph shows NDam-PolII in Fed and NR replicates. ***D–G***, Genome view of the (***D***) *moody*, (***E***) *mdr65*, (***F***) *Tret1-1*, and (***G***) *vkg* and *Col4a1* loci, showing the binding of PolII using *sema5c-GAL4* (blue track) and *mdr65-GAL4* (green track) drivers under normal Fed conditions. Note that the binding of PolII across *moody* and *mdr65*, markers of subperineurial glia, is similar in both drivers; however, the perineurial markers *Tret1-1*, *vkg* and *Col4g1* are only bound by PolII in *sema5c-GAL4* dataset. ***H***, ***I***, Diagram showing a String association network of genes downregulated in (***H***) NBs and (***I***) subperineurial glia during NR. Genes associated to the JNK pathway are shown surrounded by a gray background in ***I***. Download Figure 2-1, TIF file.

10.1523/JNEUROSCI.0452-20.2021.f2-2 Figure 2-2 Tables of genes differentially bound by PolII during Fed and NR conditions in NB, SPG, and SG TaDa. PolII binding and FDR are given for average data in Fed and NR conditions. The difference of PolII binding between Fed and NR (diff) and the ratio between them are shown. Download Figure 2-2, XLSX file.

Meta-analysis of our PolII occupancy data showed the characteristic profile of PolII binding across a gene (Southall et al., 2013; Extended Data Fig. 2-1*C*). Our TaDa replicates also correlated with each other as expected, with higher correlation among libraries of the same cell type and lower when NBs were compared with glial cells (Fig. 2*C*). Next, we analyzed the data and selected genes that were differentially bound by PolII between Fed and NR conditions in each dataset, considering genes with an FDR lower than 0.01, a PolII binding ratio higher than 2, and a difference higher than 0.3 (Fig. 2*D–F*, Extended Data Fig. 2-2). In the NB comparison, we found 14 genes upregulated and 246 genes downregulated after NR (Fig. 2*D*,*G*,*H*). In the BBB, 12 genes were upregulated and 47 downregulated in SPG (Fig. 2*E*,*I*), whereas in the SG datasets, PolII TaDa showed 44 genes upregulated and 12 genes downregulated in the NR compared with Fed condition (Fig. 2*F*,*J*). Importantly, our data showed that PolII binding to genes associated to the drivers used (*wor*, *mdr65* and *sema5c*) was not affected by NR in the corresponding experiment.

**Figure 3.**
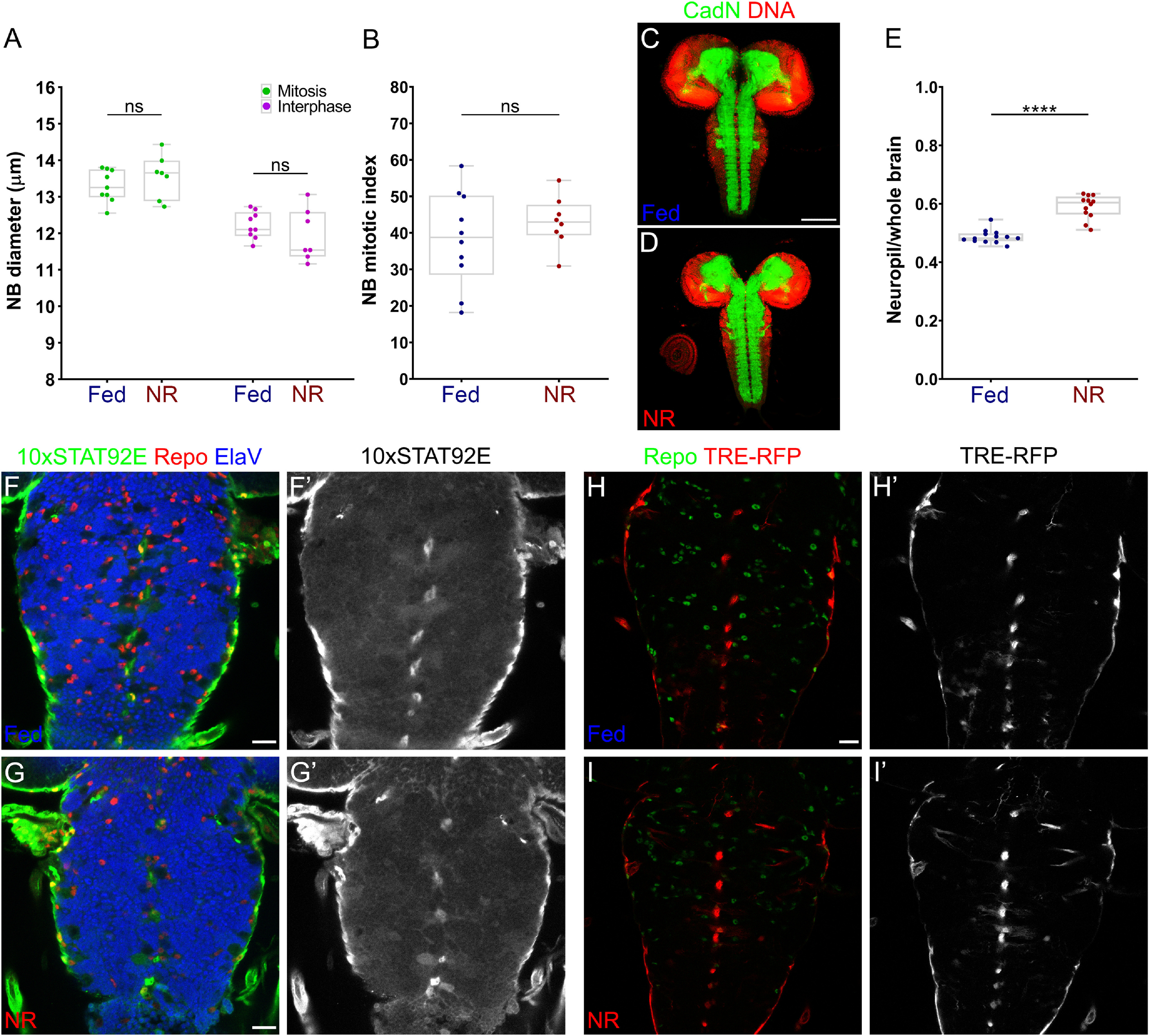
Nutrient restriction does not affect neuroblast proliferation and pathway activation in the BBB. ***A***, NB diameter during mitosis (green dots) and interphase (magenta dots) of *w^1118^* larval brains in Fed and NR conditions. *n* = 9 and 7 brain lobes, respectively. Two-way ANOVA and Bonferroni's multiple-comparisons test were performed. ns, Nonsignificant. ***B***, Graph showing the NB mitotic index of *w^1118^* larval brains under Fed and NR conditions. *n* = 10 and 8 brain lobes, respectively. Unpaired Student's *t* test was performed. ns, Nonsignificant. ***C***, ***D***, Larval CNSs immunostained for CadN (neuropil marker in green) and DNA (red) under (***C***) Fed or (***D***) NR conditions. ***E***, Plot showing the quantification of the relative size of the neuropil (neuropil/whole brain) from ***C***, ***D***. *n* = 13 and 12 CNSs, respectively. Unpaired Student's *t* test was performed, ****p < 0.0001. ***F*–*I*'**, Ventral nerve cords of (***F*–*G*'**) JAK-STAT (*10xSTAT92E-GFP*) and (***H*–*I*'**) JNK (*TRE-RFP*) pathway reporters under (***F***, ***F*'**, ***H***, ***H*'**) Fed and (***G***, ***G*'**, ***I***, ***I*'**) NR conditions. ***F–G*'**, Immunofluorescences for GFP (green and gray), Repo (red) and Elav (blue). ***H–I*'**, Immunofluorescences for Repo (green) and RFP (red and gray). Scale bars: 20 µm.

The brain-sparing model in *Drosophila* was previously shown to maintain NB proliferation on NR (Cheng et al., 2011). Our NB PolII TaDa gene ontology analysis showed that categories involved in ribosome, mitochondria, and oxidative phosphorylation metabolism were significantly enriched in downregulated genes (Fig. 2*G*, Extended Data Fig. 2-1*H*). This suggests that energy metabolism and protein synthesis were affected in neural stem cells during NR. However, we found no significant differences in the growth and proliferation of NBs between Fed and NR conditions (Fig. 3*A*,*B*), confirming that NB division and neurogenesis were not disrupted by starvation during third instar larval development (Cheng et al., 2011). In addition to this, we analyzed the neuropil of larval brains, finding that the relative size of the neuropil was increased in animals under NR (Fig. 3*C–E*), suggesting an effect of starvation over neuropil and cell body proportions.

In our BBB TaDa datasets, we found a group of proteins, associated with the JNK and Janus kinase/signal transducer and activator of transcription (JAK/STAT) signaling pathways that were downregulated after NR (Extended Data Fig. 2-1*I*). These included *Matrix metalloproteinase 1* (*Mmp1*; Uhlirova and Bohmann, 2006), *scarface* (*scaf*; Rousset et al., 2010; Srivastava and Dong, 2015), *eiger* (*egr*, TNF homolog), *TGF-*β *activated kinase 1* (*Tak1*) and the JAK/STAT ligands *unpaired 1* (*upd1*), *unpaired 2* (*upd2*), and *unpaired 3* (*upd3*; Fig. 2*I*,*J*, Extended Data Fig. 2-1*I*). Therefore, we analyzed whether the JAK/STAT and JNK pathways were active in the larval BBB and if their activity was modulated by larval nutrition. We used the *10xSTAT92E-GFP* (Bach et al., 2007) and *TRE-RFP* (Chatterjee and Bohmann, 2012) reporters to evaluate JAK/STAT and JNK pathway activity, respectively. As expected, both reporters were expressed in the BBB during larval development; however, the expression of these reporters did not change after nutrient restriction (Fig. 3*F–I*'), suggesting that these signaling pathways in the BBB are insensitive (not responding) to the nutritional status of the animal.

### Nutrient restriction affects the development of the *Drosophila* blood–brain barrier

Because NB growth and proliferation were not affected by NR, we hypothesized that the larval BBB might adapt to starvation, being the first layer of the brain to sense and respond to a decrease in nutrients in the larval hemolymph.

**Figure 4.**
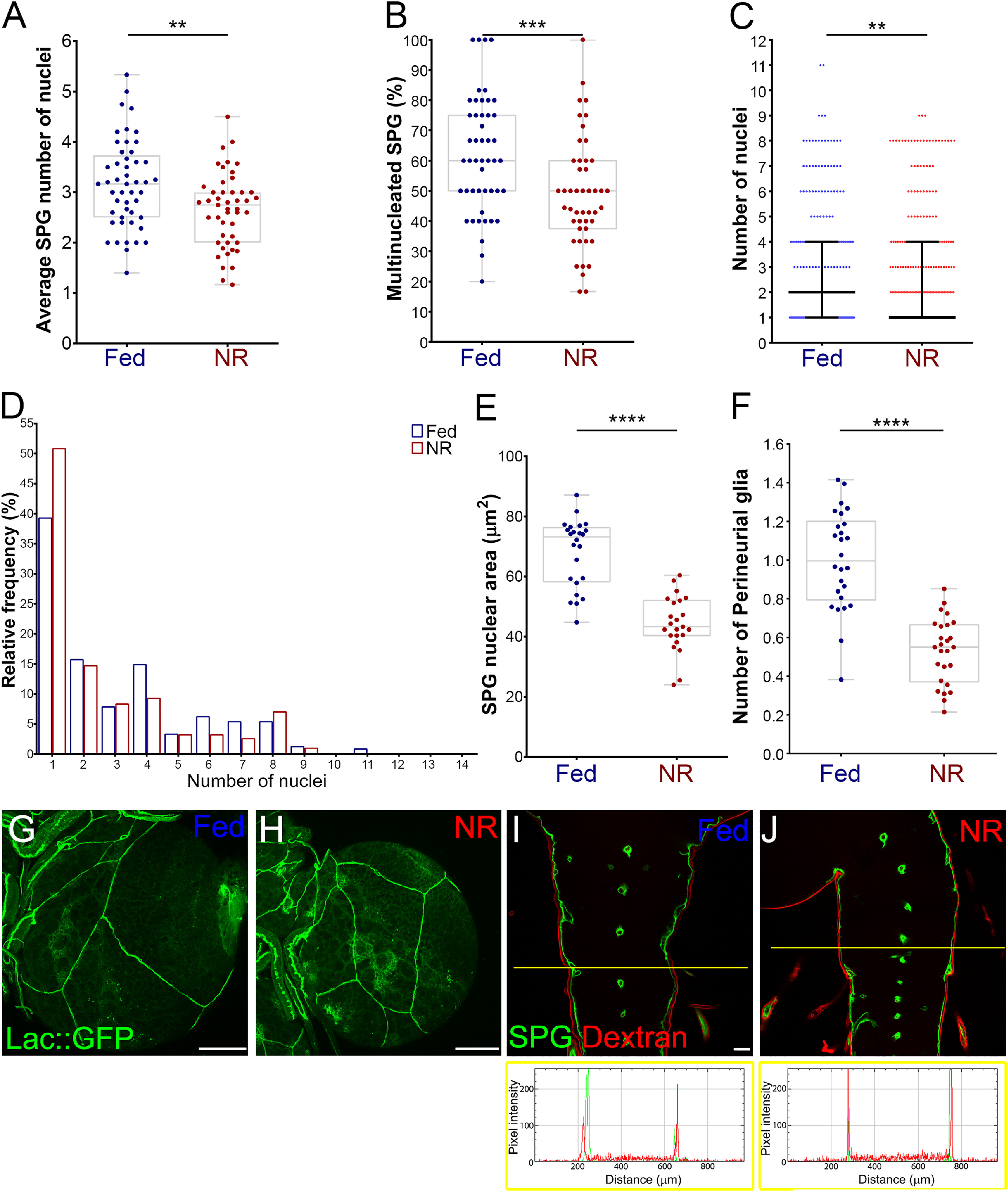
Nutrient restriction affects the growth of the blood–brain barrier. ***A–D***, Analysis of larval SPG endomitosis of animals (*mdr65-GAL4, UAS-DlgA::GFP*) in Fed and NR conditions. ***A***, Graph showing the average number of SPG nuclei per brain lobe. *n* = 49 and 47 brain lobes. Unpaired Student's *t* test was performed. ***B***, Plot showing the percentage of multinucleated SPG (2 or more nuclei) per brain lobe. *n* = 48 and 47 brain lobes, respectively. Mann–Whitney test was done. ***C***, Plot showing the number of nuclei in each SPG analyzed, median (black line) and interquartile range are shown. Mann–Whitney test was done, Fed and NR *n* = 242 and 313 SPG, respectively. ***D***, Histogram depicting the relative distribution (percentage) of SPG according to the number of nuclei. ***E***, Graph showing the average size of the SPG nucleus (*moody-GAL4, UAS-GFP.nls*) of larval brains in Fed and NR conditions. *n* = 24 and 23 brain lobes, respectively. Unpaired Student's *t* test was used for *p* value. ***F***, Plot showing the distribution of the number of PG per brain lobe. *moody-GAL4, UAS-GFP.nls* were stained for the glial marker Repo, and PG were scored as Repo-positive/GFP-negative nuclei. *n* = 26 brain lobes for Fed and NR conditions. Unpaired Student's *t* test was used. ***G***, ***H***, Larval brain lobes of *lac::GFP* animals under (***G***) Fed and (***H***) NR conditions, showing the integrity of septate junctions. Scale bars: 50 µm. ***I***, ***J***, Blood–brain barrier permeability assay in *mdr65-GAL4, UAS-mCD8-GFP* animals under (***I***) Fed or (***J***) NR conditions. Images show the ventral nerve cord, SPG membrane in green and 10 kDa dextran-Rhodamine in red. Plots show the intensity of fluorophores across the yellow line in each image. Scale bar, 20 µm. **p < 0.01, ***p < 0.01, ****p < 0.0001.

The two types of glial cells that form the BBB, the SPG and PG have different mechanisms of growth. SPG grow by endoreplication resulting in polyploid cells (Unhavaithaya and Orr-Weaver, 2012), whereas PG proliferate, undergoing mitosis (Pereanu et al., 2005; Awasaki et al., 2008; Avet-Rochex et al., 2012). SPG endoreplication takes place by either endocycle or endomitosis (Unhavaithaya and Orr-Weaver, 2012; Øvrebø and Edgar, 2018). Therefore, we checked the SPG for endomitosis by counting the number of SPG nuclei in third instar larval brains subjected to NR. We found a significant reduction in the number of nuclei in SPG (Fig. 4*A*) and a significant decrease in the percentage of multinucleated SPG (Fig. 4*B*). Similarly, the distribution of SPG with multiple nuclei changed significantly from a median of two in Fed animals to one after NR (Fig. 4*C*,*D*). Given that the size of the nucleus correlates highly with cellular size and DNA content (Frawley and Orr-Weaver, 2015), nuclear size was measured as an indicator of the SPG endocycle. We found a significant reduction in average nuclear size after NR (Fig. 4*E*). We also counted the number of PG on NR to check PG proliferation and observed a reduction in PG proliferation (Fig. 4*F*). These results confirmed a major effect of nutrition on the BBB glial cells—a reduction in SPG endoreplication and a decrease in PG proliferation after food deprivation.

A severe reduction in SPG endoreplication affects the maintenance of the subperineurial septate junctions, allowing paracellular diffusion inside the larval brain (Unhavaithaya and Orr-Weaver, 2012; Von Stetina et al., 2018). Therefore, we checked the integrity of SPG septate junctions after nutrient restriction using an endogenously tagged version of the septate junction structural protein *lachesin* (*lac::GFP*; Morin et al., 2001). We found no major disruptions in the continuity of the septate junctions in larval brains under NR conditions (Fig. 4*G–H*). To test further the integrity of the BBB, we assessed BBB permeability by incubating larval brains with rhodamine-labeled dextran (10 kDa) and assayed for penetration inside the ventral nerve cord. Rhodamine-dextran did not enter the CNS and remained on top of the subperineurial layer, marked by *mdr65-GAL4, UAS-mCD8-GFP* (Fig. 4*I*,*J*). These results suggest that the integrity of the larval BBB is not disrupted by larval NR, despite the reduction in BBB growth.

### Scarface is expressed in subperineurial glia and is regulated by nutrient availability

**Figure 5.**
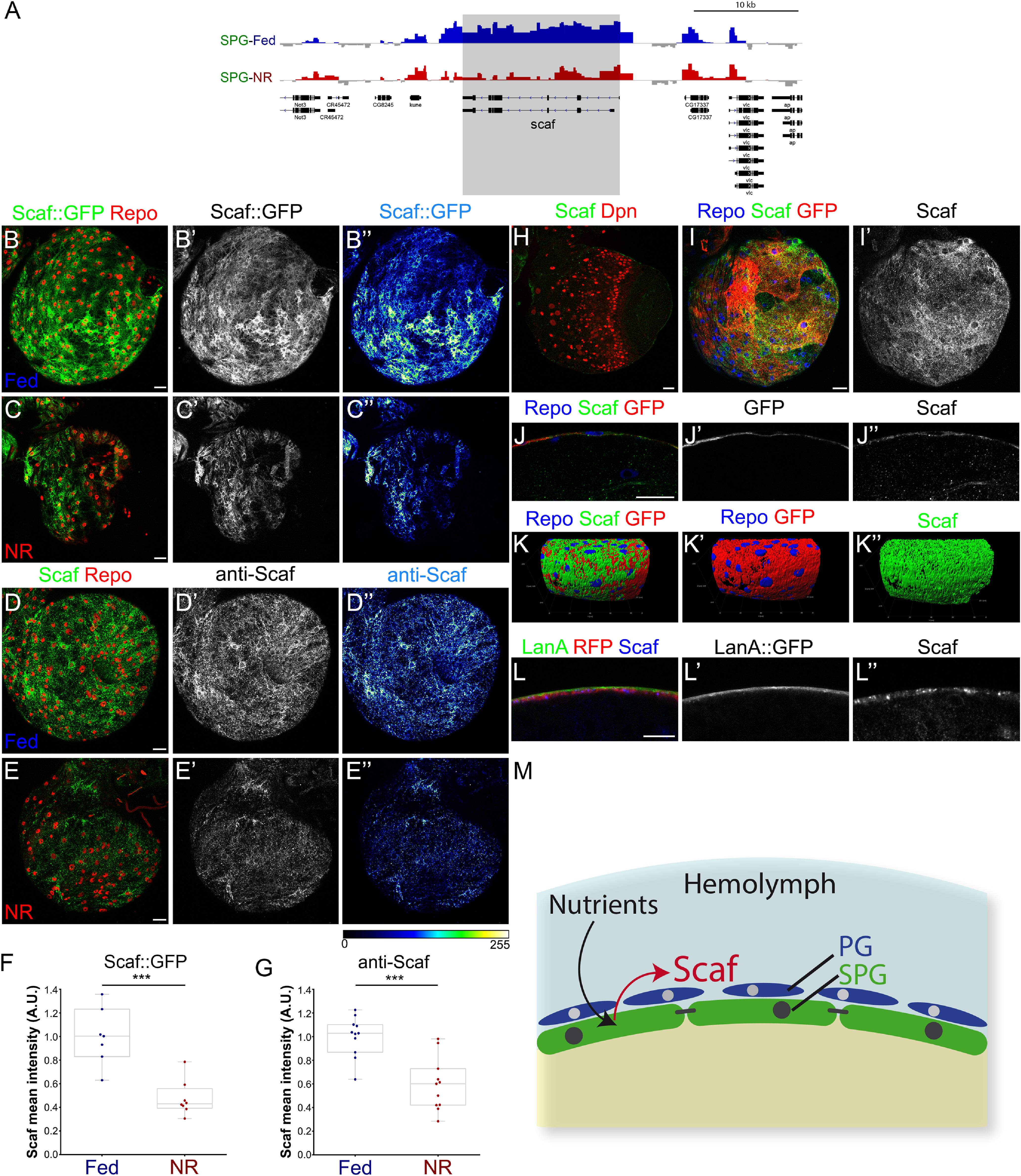
Scarface is a nutrient-sensitive gene expressed in the *Drosophila* blood–brain barrier. ***A***, Genome view of the *scaf* locus and the binding of PolII in SPG TaDa in Fed (blue) and NR (red) conditions. Note the reduction on PolII binding in NR compared with Fed. ***B–C*''**, Immunostaining of *scaf::GFP* (*scaf^PBss^*, in green) larval brain lobes (optical section at the BBB level) under (***B–B*''**) Fed and (***C–C*''**) NR conditions stained for Repo (glial marker in red). ***B*''**, ***C*''**, are color-coded images of Scaf::GFP signal. ***D–E*''**, Immunostaining of larval brains under (***D–D*''**) Fed and (***E–E*''**) NR conditions stained for Scaf (green) and Repo (red). ***D*''**, ***E*''** are color-coded images of anti-Scaf antibody signal. ***F***, ***G***, Graph showing the quantification of the Scaf signal in the BBB using (***F***) Scaf::GFP reporter (*n* = 7 and 8 brains) or (***G***) anti-Scaf antibody (*n* = 11 brains). Unpaired Student's *t* test was used, ***p < 0.001. ***H***, Confocal images of a larval brain stained with anti-Scaf (green) and Dpn (NB marker in red). ***I***, ***I*'**, Larval brain expressing mCD8GFP in SPG (*mdr65-Gal4, UASmCD8-GFP*, in red) and stained for Repo (glial marker in blue) and Scaf (green or gray). ***J–J*''**, Cross section of larval BBB of *mdr65-GAL4, UAS-mCD8GFP* animals stained against Repo (blue), Scaf (green or gray) and mCD8-GFP (red or gray). ***K–K*''**, 3D reconstruction of the larval BBB of *mdr65-GAL4, UAS-mCD8GFP* animals stained against Repo (blue), Scaf (green), and mCD8-GFP (red). ***L–L*''**, Representative confocal image of a cross section of a larval brain stained against Scaf (blue and gray), *sema5-GAL4, UAS-mCD8-RFP* (red) and LanA::GFP (green and gray) as a neural lamella marker. Scale bars, ***B–E*''**, ***H–J*''**, 20 µm; ***L–L*''**, 0 µm. ***M***, Graphic representation of the regulation of *scaf* expression in the blood–brain barrier by nutrition.

Because the growth of the BBB is affected by NR, we focused on BBB genes that were differentially expressed as indicated by our TaDa analysis. Of those, we centered our interest on *scarface* (*scaf*), one of the most downregulated genes during NR in SPG (Figs. 2*I*, 5*A*). Scaf belongs to the SPH family (Ross et al., 2003; Bonin and Mann, 2004; Rousset et al., 2010; Sorrosal et al., 2010). Serine protease homologs resemble proteases but lack the amino acids required for enzymatic activity. Scaf controls epithelial polarity and morphogenesis during embryonic development and thorax formation, acting as a downstream target of the JNK pathway, and it has been reported to be a secreted protein (Rousset et al., 2010; Sorrosal et al., 2010; Srivastava and Dong, 2015; Kushnir et al., 2017).

To validate that *scaf* was differentially expressed on NR, we made use of a gene trap line (*scaf^PBss^* allele, Scaf::GFP; Bonin and Mann, 2004) and an antibody against Scaf (anti-Scaf; Rousset et al., 2010). Although, the *scaf^PBss^* is a semilethal allele, when used in heterozygosis it is a reliable tool for analyzing *scaf* expression. We observed Scaf expression in glial cells at the surface of the larval brain, corresponding to the BBB (Fig. 5*B–B*'', *D–D*''). As predicted by TaDa, the levels of Scaf were reduced after NR (Fig. 5*C–C*'', *E–E*''). Intensity quantification of the Scaf signal showed a significant reduction of an average of 47.56% and 59.37% of the Scaf intensity of Fed animals (Scaf::GFP and anti-Scaf, respectively; Fig. 5*F*,*G*).

As predicted by our SPG TaDa analysis, Scaf colocalised with an SPG membrane marker (*mdr65-GAL4, UAS-mCD8-GFP*, Fig. 5*I–J*'') at the surface of the brain, but not with NBs, labeled by Dpn (Fig. 5*H*). Interestingly, the Scaf signal appeared also on top of the SPG membrane marker in a 3D reconstruction (Fig. 5*K–K*''). To confirm this, we used an endogenously tagged *Laminin A* (*LanA::GFP*) line to label the brain extracellular matrix (neural lamella) and an SG membrane marker (*sema5c-GAL4, UAS-mCD8-RFP*). Because the thickness of the BBB is ∼2 µm, we observed that the Scaf signal overlapped with LanA and most of SG membrane signals, thus the entire BBB (Fig. 5*L–L*''). These results showed that during third instar larval development *scaf* is expressed by SPG in a nutrition-sensitive fashion (Fig. 5*M*).

### Scarface reduces the growth of the blood–brain barrier during larval development

**Figure 6.**
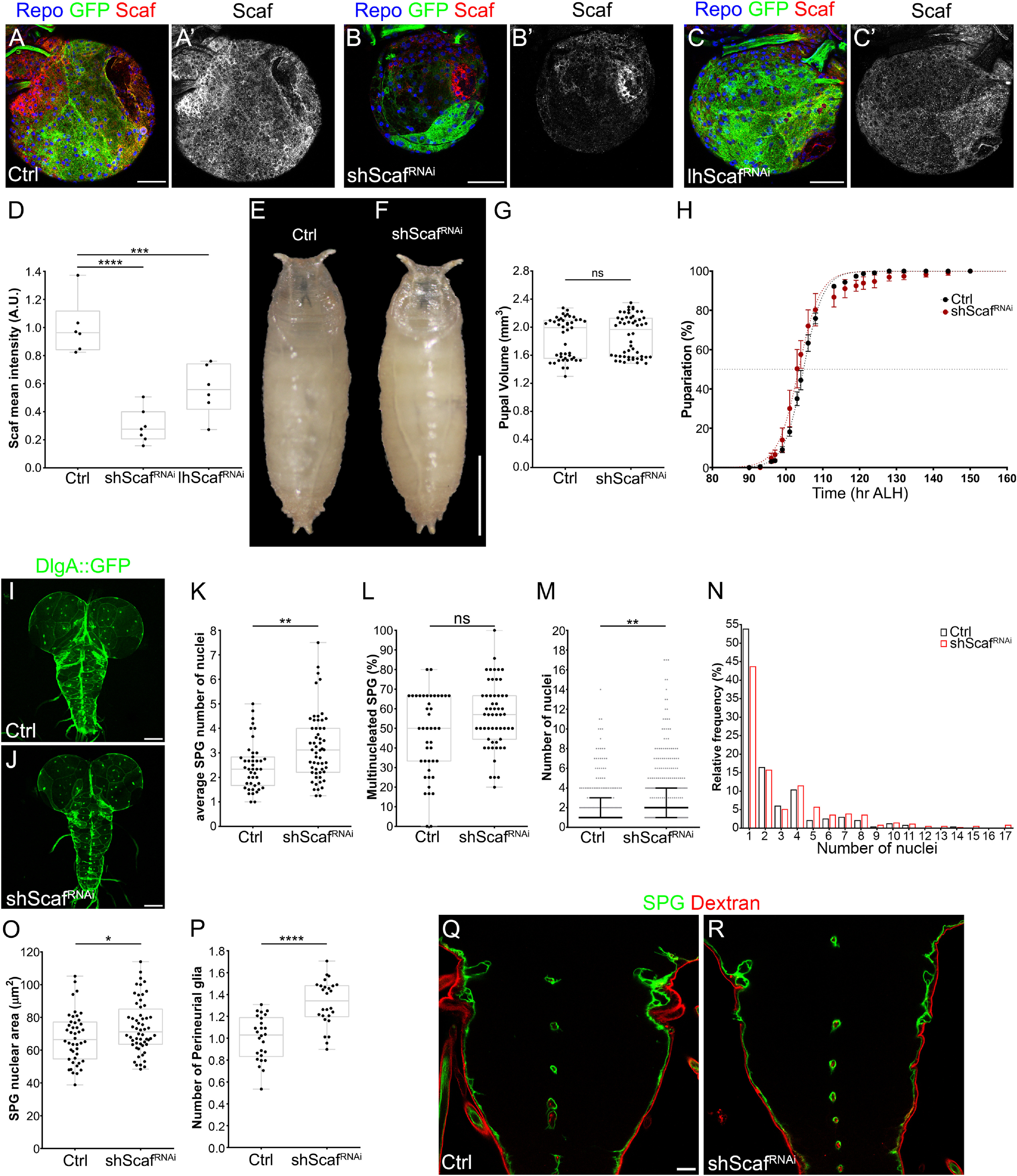
Scarface is necessary for controlling the growth of the blood–brain barrier. ***A–C*'**, Immunostaining of larval brains of *mdr65-GAL4, UAS-mCD8GFP* animals crossed to (***A***, ***A*'**) *w^1118^* (control), (***B***, ***B*'**) *UAS-shScaf^RNAi^* and (***C***, ***C*'**) *UAS-lhScaf^RNAi^*, and stained for Repo (blue), Scaf (red and gray) and GFP (green). Scale bars: 50 µm. (***D***) Plot showing the quantification of *scaf* knockdown. *n* = 6, 7, 6 brains, respectively. One-way ANOVA and Dunnett's multiple comparisons test were performed. ***E***, ***F***, Images of pupae of *mdr65-GAL4* crossed to (***E***) *w^1118^* (control) and (***F***) *UAS-shScaf^RNAi^*. ***G***, Plot showing the pupal volume for each group. *n* = 45 and 52 pupae, respectively. Mann–Whitney test was performed. ***H***, Graph showing the timing of pupariation of *mdr65-GAL4* crossed to *w^1118^* (Ctrl), dark or *UAS-shScaf^RNAi^* (red). *n* = 6 groups of ∼35–40 larvae for each condition. ***I***, ***J***, Larval CNS of *mdr65-GAL4, UAS-DlgA:EGFP* animals crossed to (***I***) *w^1118^* (control) and (***J***) *UAS-shScaf^RNAi^*. *UAS-DlgA:EGFP* labels SPG nuclei and septate junction. Scale bars: 100 µm. ***K–N***, Analysis of larval SPG endomitosis. *mdr65-GAL4, UAS-DlgA::GFP* animals were crossed to *w^1118^* (control) and *scaf* knockdown (*UAS-shScaf^RNAi^*). ***K***, Graph showing the average number of SPG nuclei per brain lobe. *n* = 43 and 46 brain lobes. Unpaired Student's *t* test was performed. ***L***, Plot showing the percentage of multinucleated SPG (2 or more nuclei) per brain lobe. *n* = 43 and 56 brain lobes. Mann–Whitney test was done. ***M***, Plot showing the number of nuclei in each SPG analyzed, median (black line) and interquartile range are shown. Mann–Whitney test was done, *n* = 230 (Ctrl) 320 (shScaf^RNAi^) SPG. ***N***, Histogram showing the relative distribution (percentage) of SPG according to the number of nuclei. ***O***, Graph showing the average size of the SPG nucleus of larval brains. *mdr65-GAL4, UAS-DlgA::GFP* animals were crossed to *w^1118^* (control) and *UAS-shScaf^RNAi^*. *n* = 44 and 56 brain lobes, respectively. Unpaired Student's *t* test was used. ***P***, Plot showing the distribution of the number of PG per brain lobe. *mdr65-GAL4, UAS-DlgA::GFP* were stained for the glial marker Repo, Repo-positive/GFP-negative nuclei were counted as PG. *n* = 26 brains for both condition. Unpaired Student's *t* test was performed. ***Q***, ***R***, Blood–brain barrier permeability assay in *mdr65-GAL4, UAS-mCD8-GFP* animals crossed to (***Q***) *w^1118^* (control) or (***R***) *UAS-shScaf^RNAi^*. Images show the ventral nerve cord, SPG membrane in green and 10 kDa dextran-Rhodamine in red. *p <0.05, **p < 0.01, ***p < 0.001, ****p < 0.0001, respectively. ns, Nonsignificant. *scaf* knockdown using UAS-lh*Scaf^RNAi^* is shown in Extended Data Figure 6-1.

As SPG expression of Scaf is sensitive to nutrition, we aimed to assess its function in the BBB during normal development. To accomplish this, we performed RNAi-mediated knockdown of *scaf* only in SPG using the *mdr65-GAL4* driver. We tested two different RNAi lines, a short hairpin (*shScaf^RNAi^*) and a long hairpin (*lhScaf^RNAi^*), finding that both were able to significantly reduce the levels of Scaf in the BBB (Fig. 6*A–D*). Because *shScaf^RNAi^* gave the most efficient knockdown (29.63% of control Scaf levels), we used this line in most of our experiments.

10.1523/JNEUROSCI.0452-20.2021.f6-1 Figure 6-1 ***A–D***, Analysis of larval SPG endomitosis of *mdr65-GAL4, UAS-DlgA::GFP* animals crossed to *w^1118^* (control) and *UAS-lhScaf^RNAi^* (*scaf* knockdown). ***A***, Graph showing the average number of SPG nuclei per brain lobe. *n* = 60 and 37 brain lobes. Unpaired Student's *t* test was performed. ***B***, Plot showing the percentage of multinucleated SPG (2 or more nuclei) per brain lobe. *n* = 60 and 37 brain lobes. Mann–Whitney test was done. ***C***, Plot showing the number of nuclei in each SPG analyzed, median (black line) and interquartile range are shown. Mann–Whitney test was done,. *n* = 374 and 189 SPG for control (Ctrl) and lhScaf^RNAi^, respectively. ***D***, Histogram depicting the relative distribution (percentage) of SPG according to the number of nuclei. ***E***, Graph showing the average size of the SPG nucleus of larval brains of *mdr-GAL4, UAS-DlgA::GFP* animals crossed to *w^1118^* (control) and *UAS-lhScaf^RNAi^*. *n* = 30 and 27 brain lobes, respectively. Unpaired Student's *t* test was used. ***F***, Plot showing the distribution of the number of PG per brain lobe. *mdr65-GAL4, UAS-GFP.nls* were crossed to *w^1118^* (control) and *UAS-lhScaf^RNAi^* and stained for the glial marker Repo. Repo-positive/GFP-negative nuclei were scored as PG. *n* = 24 and 26 brain lobes. Unpaired Student's *t* test was used. *p < 0.05, **p < 0.01, ***p < 0.001, ****p < 0.0001. Download Figure 6-1, TIF file.

Knockdown of *scaf* in SPG produced normal larvae, which grew and pupariated as control animals and emerged into fertile adult flies (Fig. 6*E–H*). We checked the development of the BBB at late third instar larval stage, observing no morphologic defects (Fig. 6*I*,*J*). Thus, we analyzed endomitosis of SPG, finding a significant increase in the number of SPG nuclei after *scaf* knockdown (Fig. 6*K*) but without a significant change in the percentage of multinucleated SPG per brain lobe (Fig. 6*L*). This suggested that on *scaf* knockdown, the number of multinucleated SPG is similar, but they contain more nuclei than in control animals. This was confirmed by analyzing the distribution of SPG according to the number of their nuclei, showing an increase in the median from one nucleus in control animals to two nuclei in *shScaf^RNAi^* brains (Fig. 6*M*,*N*).

Next, we checked the nuclear size in SPG, observing a significant increase on *scaf* knockdown (Fig. 6*O*), which suggests an increase in SPG endocycle. We also found a significant increase in the number of PG in *shScaf^RNAi^* animals (Fig. 6*P*). We confirmed these results using the *lhScaf^RNAi^* strain (Extended Data Fig. 6-1). Next, we assessed whether permeability of the BBB was altered in *scaf* knockdown larval brains using the Rhodamine-dextran assay described before. We did not detect the colorant inside the CNS (Fig. 6*Q*,*R*), ratifying that a reduction in the levels of Scaf increases the growth of the subperineurial layer as well as the number of PG without affecting the BBB permeability.

**Figure 7.**
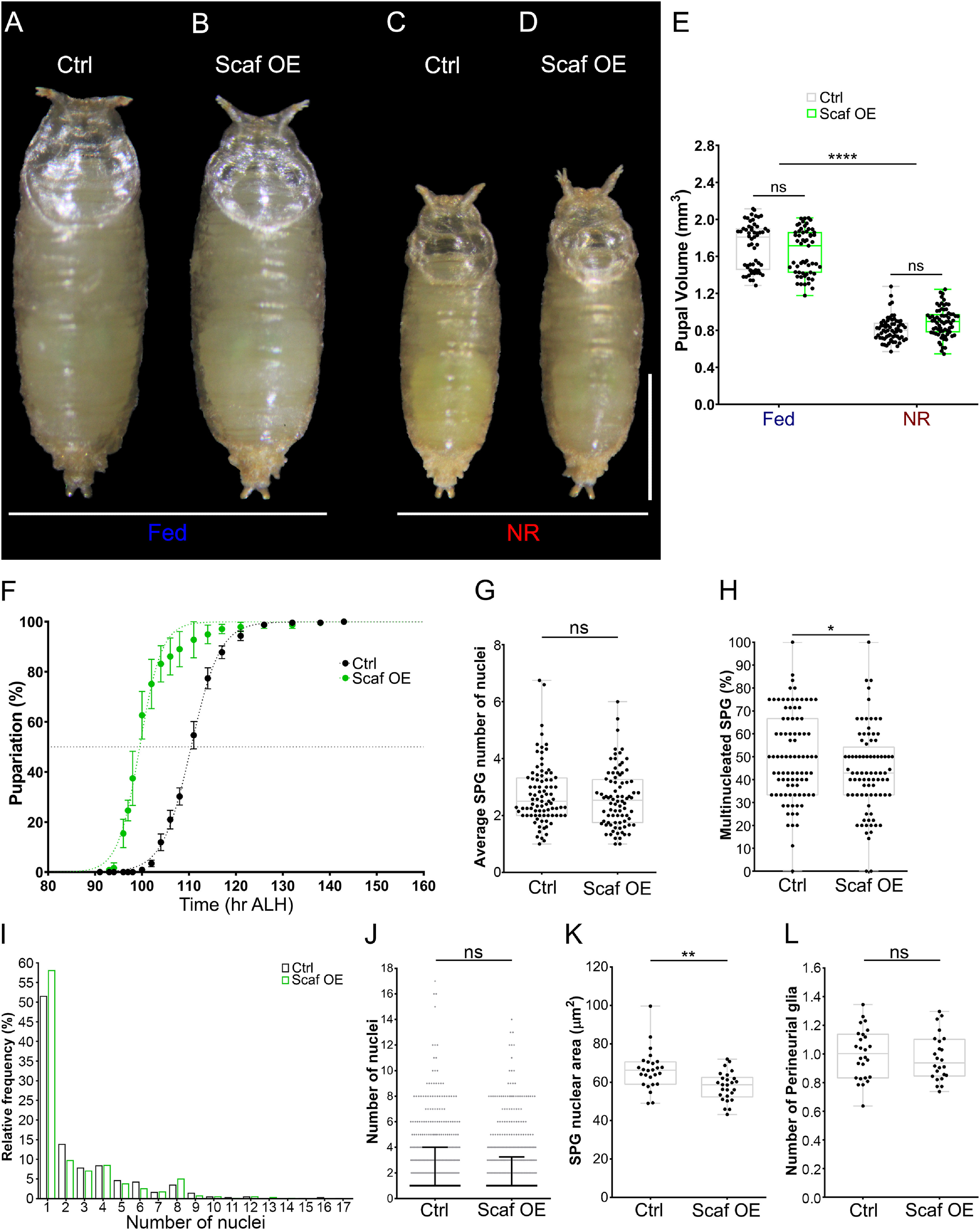
Scarface overexpression in subperineurial glia affects larval development and the growth of the BBB. ***A–D***, Images of pupae of *mdr65-GAL4* crossed to (***A***, ***C***) *w^1118^* or (***B***, ***D***) *UAS-Scaf::GFP* (Scaf OE) under (***A***, ***B***) Fed or (***C***, ***D***) NR conditions. Scale bar, 1 mm. ***E***, Graph showing the pupal volume. *n* = 57, 57, 67 and 70 pupae, respectively. Two-way ANOVA and Bonferroni's multiple-comparisons tests were done. ***F***, Graph showing the timing of pupariation of *mdr65-GAL4* crossed to *w^1118^* (Ctrl), dark or *UAS-Scaf::GFP* (Scaf OE, green). *n* = 6 groups of ∼35–40 larvae for each condition. ***G–J***, Analysis of larval SPG endomitosis of *mdr65-GAL4, UAS-DlgA::GFP* animals crossed to *w^1118^* (control) and *UAS-Scaf::GFP* (Scaf OE). ***G***, Graph showing the average number of SPG nuclei per brain lobe. *n* = 88 and 84 brain lobes. Unpaired Student's *t* test was performed. ***H***, Plot showing the percentage of multinucleated SPG (2 or more nuclei) per brain lobe. *n* = 89 and 84 brain lobes. Mann–Whitney test was done. ***I***, Histogram showing the relative distribution (percentage) of SPG according to the number of nuclei. ***J***, Plot showing the number of nuclei in each SPG analyzed, median (black bar) and interquartile range are shown. Mann–Whitney test was done, *n* = 531 (Ctrl) and 440 (Scaf OE) SPG. ***K***, Plot showing of the average size of SPG nuclei. *moody-GAL4, UAS-GFP.nls* was crossed to *w^1118^* (control) and *UAS-Scaf::GFP* (Scaf OE). *n* = 26 brain lobes for each condition. Unpaired Student's *t* test was used. ***L***, Plot showing the distribution of the number of PG in brain lobe. *moody-GAL4, UAS-GFP.nls* were stained for the glial marker Repo, Repo-positive/GFP-negative nuclei were scored as PG. *n* = 26 and 24 brain lobes. Unpaired Student's *t* test was used. *p < 0.05, **p < 0.01, ***p < 0.001, ****p < 0.0001. ns, Nonsignificant.

Given that *scaf* knockdown augmented the growth of the BBB, increasing the levels of Scaf should produce the opposite effect. To perform gain of function experiments in SPG during development, we used a line (*UAS-Scaf*) that rescues *scaf* mutant embryonic lethality (Rousset et al., 2010). Overexpression of Scaf generated normal and viable animals, without affecting larval growth under Fed or NR conditions. However, the timing of puparation was significantly accelerated (see Fig. 7*A–F*). In the BBB, we found that the average number of SPG nuclei did not change on Scaf overexpression (Fig. 7*G*); however, the percentage of multinucleated SPG in a brain lobe was significantly decreased (Fig. 7*H*). We also observed an increase in the mononucleated fraction of SPG but without a change in the overall distribution (Fig. 7*I*,*J*). Interestingly, the size of the SPG nuclei was significantly reduced by Scaf overexpression (Fig. 7*K*). These results showed that increasing the levels of Scaf has a mild effect on SPG endoreplication. In the perineurial layer, the number of PG was not affected by Scaf overexpression (Fig. 7*L*), suggesting that there was no impact on PG proliferation. Our data infer a role for Scaf in diminishing the growth of the BBB through decreasing the levels of the SPG endocycle and endomitosis during development under normal nutritional conditions.

### Blood–brain barrier–derived scarface promotes neurogenesis in the larval brain

**Figure 8.**
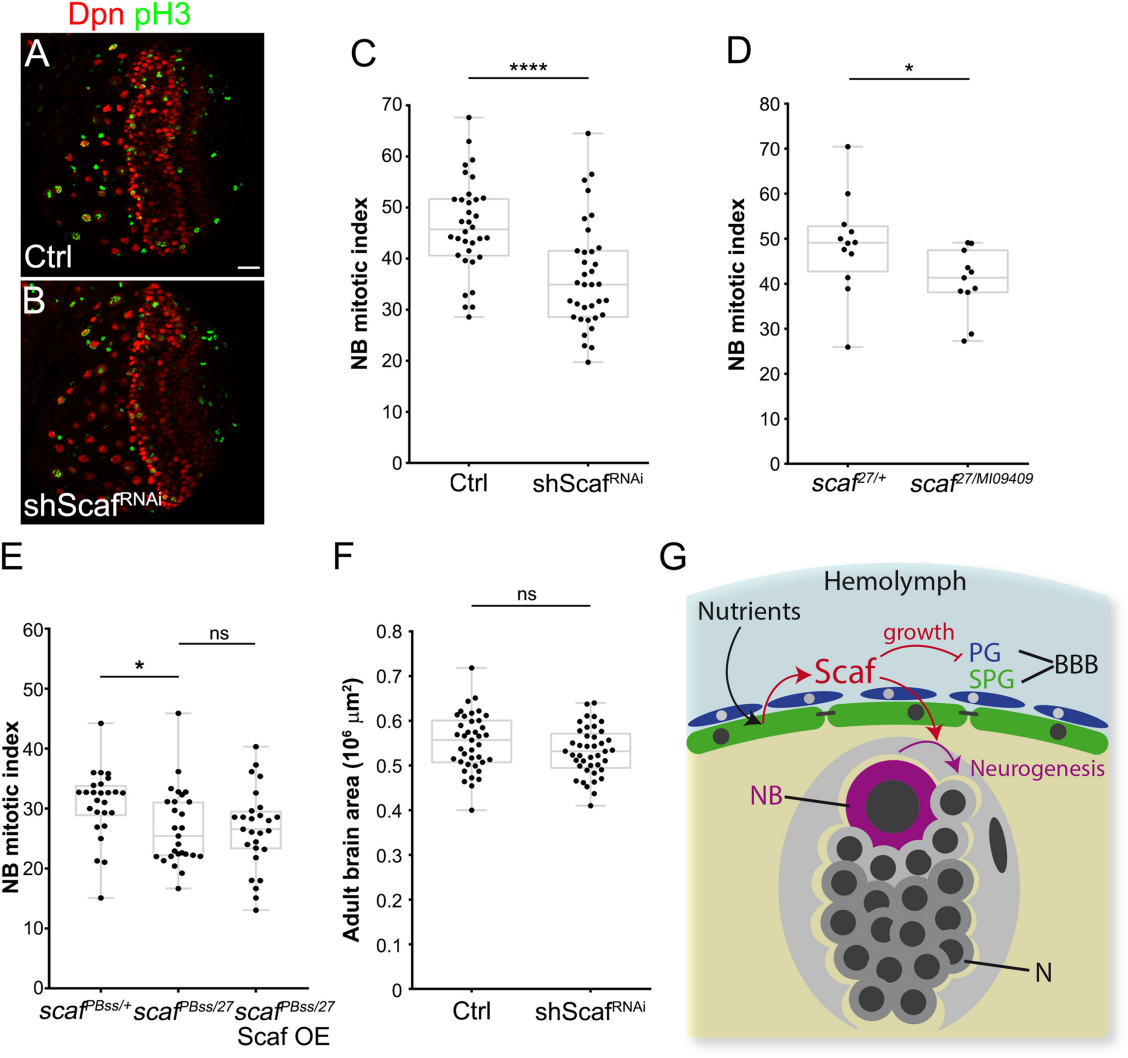
Scarface modulates neurogenesis in the larval brain. ***A***, ***B***, Immunostaining of larval brains of *mdr65-GAL4* crossed to (***A***) *w^1118^* (control) and (***B***) *UAS-shScaf^RNAi^*, stained for the NB marker Dpn (red) and phospho-Histone H3 (pH3 in green). Scale bar, 20 µm. ***C***, Graph showing the NB mitotic index (percentage of pH3-positive NB) of control and *scaf* knockdown brains. *n* = 34 and 35 brain lobes, respectively. Unpaired Student's *t* test was performed. ***D***, Graph showing the NB mitotic index of *scaf^27/+^* (heterozygous) and *scaf^27/MI09409^*. *n* = 12 and 11 brain lobes, respectively. Unpaired Student's *t* test was performed. ***E***, NB mitotic index of *scaf^PBss/+^*, *mdr65-GAL4* (heterozygous); *scaf^PBss/27^*, *mdr65-GAL4*; and *scaf^PBss/27^*; *mdr65-GAL4*, *UAS-Scaf:GFP* animals. *n* = 26, 27, and 27 brain lobes, respectively. One-way ANOVA and Dunnett's multiple-comparisons test were performed. ***F***, Plot of the size of the adult brain of *mdr65-GAL4* crossed to *w^1118^* (control) and *UAS-shScaf^RNAi^*. *n* = 39 and 41 adult brains respectively. Unpaired Student's *t* test was performed. *p < 0.05, ****p < 0.0001. ns, Nonsignificant. ***G***, Working model of Scaf function in the *Drosophila* blood–brain barrier. As a response to nutrient availability, the subperineurial glial cells in the BBB express Scaf. Scaf slows down the growth of SPG and diminishes the proliferation of PG but at the same time promotes NB proliferation.

Nutrient restriction reduced the levels of Scaf in the BBB and also produced animals with smaller brains than the control Fed condition. We assessed whether Scaf function at the BBB has a nonautonomous effect on neurogenesis. Thus, we knocked down *scaf* in SPG and checked the mitotic index of larval central brain NBs, observing a significant decrease in the fraction of NBs in mitosis (Fig. 8*A–C*). To confirm this result, we used a *scaf* allelic combination of *scaf^27^*, a deletion resulting in a strong lethal allele (Rousset et al., 2010), with *scaf^MI09409^*, a semilethal allele generated by a MiMIC insertion (Venken et al., 2011), producing a truncated Scaf protein (that retains the antibody epitope). This, *scaf^27^*/*scaf^MI09409^* allelic combination is a hypomorphic condition that is viable and healthy during larval development but lethal at late pupal stage. Similar to *scaf* knockdown experiments, we found that *scaf* mutant brains had a significantly lower NB mitotic index than heterozygous animals (*scaf^27/+^*; Fig. 8*D*). In the same way, the hypomorphic allelic combination, *scaf^PBss^*/*scaf^27^*, also showed a decreased NB mitotic index compared with the heterozygous control (*scaf^PBss^*^/+^; Fig. 8*E*,*G*). Because *scaf* in the BBB was necessary for proper NB proliferation, we conducted a rescue experiment to determine whether the expression of Scaf only in SPG was sufficient to maintain the normal NB mitotic index in *scaf* mutant animals. As seen in Figure 8*E*, the overexpression of *scaf* was not able to rescue NB mitotic index in a *scaf* mutant background. Thus, Scaf from SPG is not sufficient to maintain NB proliferation in the mutant (Fig. 8*E*). Additionally, the knockdown of *scaf* only in SPG did not have a major impact on the overall size of the brain (Fig. 8*F*). These results suggest that Scaf is necessary in the BBB to maintain the rate of larval neurogenesis, but Scaf in other cell types is also required for NB proliferation.

**Figure 9.**
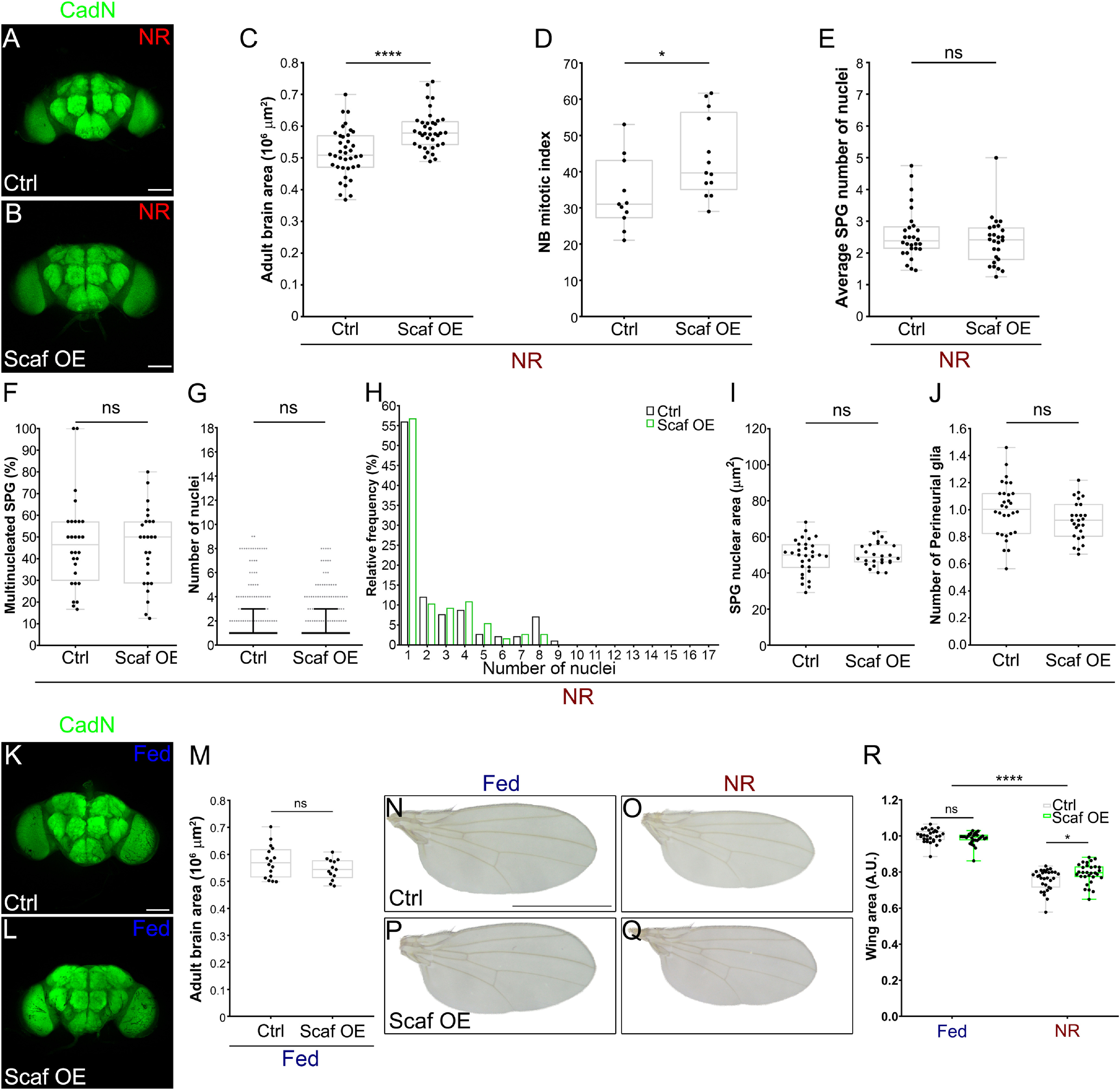
Expression of Scarface in subperineurial glia increases neurogenesis during nutrient restriction. ***A***, ***B***, Adult brains stained for CadN (green) of *mdr65-GAL4* crossed to (***A***) *w^1118^* (control) and (***B***) *UAS-Scaf::GFP* (Scaf OE) under NR. Scale bar, 100 µm. ***C***, Quantification of the adult brain area. *mdr65-GAL4* animals were crossed to *w^1118^* (control) and *UAS-Scaf::GFP* (Scaf OE) under NR. *n* = 38 and 37 adult brains, respectively. Unpaired Student's *t* test was performed. ***D***, Graph of the NB mitotic index of *mdr65-GAL4* animals crossed to *w^1118^* (control) and *UAS-Scaf::GFP* (Scaf OE) larval brains under NR. *n* = 11 and 13 brain lobes, respectively. Unpaired Student's *t* test was performed. ***E–H***, Analysis of larval SPG endomitosis of *mdr65-GAL4, UAS-DlgA::GFP* animals crossed to *w^1118^* (control) and *scaf* overexpression (*UAS-Scaf::GFP*, Scaf OE) under NR. ***E***, Graph showing the average number of SPG nuclei per brain lobe. *n* = 28 and 26 brain lobes. Unpaired Student's *t* test was performed. ***F***, Plot showing the percentage of multinucleated SPG (2 or more nuclei) per brain lobe. *n* = 28 and 26 brain lobes. Mann–Whitney test was used. ***G***, Plot showing the number of nuclei in each SPG analyzed, median (black line) and interquartile range are shown. Mann–Whitney test was performed. *n* = 182 (Ctrl) and 183 (Scaf OE) SPG. ***H***, Histogram depicting the relative distribution (percentage) of SPG according to the number of nuclei. ***I***, Graph showing the average size of the SPG nucleus of larval brains of *moody-GAL4, UAS-GFP.nls* animals crossed to *w^1118^* (control) and Scaf overexpression (*UAS-Scaf::GFP*, Scaf OE) under NR. *n* = 30 and 26 brain lobes. Unpaired Student's *t* test was used. ***J***, Plot showing the distribution of the number of PG per brain lobe. *moody-GAL4, UAS-GFP.nls* were crossed to *w^1118^* (control) and Scaf overexpression (*UAS-Scaf::GFP*, Scaf OE) under NR, and stained for the glial marker Repo. Repo-positive/GFP-negative nuclei were scored as PG. *n* = 30 and 26 brain lobes. Unpaired Student's *t* test was used. ***K***, ***L***, Immunostaining against CadN of adult female brains of *mdr65-GAL4* crossed to (***K***) *w^1118^* (Ctrl) or (***L***) *UAS-Scaf::GFP* (Scaf OE). Scale bar, 100 µm. ***M***, Plot showing the size of the adult brain of *mdr65-GAL4* crossed to *w^1118^* (control) and *UAS-Scaf::GFP* (Scaf OE). *n* = 16 and 13 adult brains, respectively. Unpaired Student's *t* test was performed. ***N–Q***, Wings of male animals of *mdr65-GAL4* crossed to (***N***, ***O***) *w^1118^* (Ctrl) and (***P***, ***Q***) *UAS-Scaf::GFP* (Scaf OE) under (***N***, ***P***) Fed or (***O***, ***Q***) NR conditions. Scale bar, 1 mm. ***R***, Plot showing the quantification of the size of the wings for each conditions. *n* = 29, 30, 30, and 30 wings, respectively. Two-way ANOVA and Tukey's multiple-comparison tests were done. *p < 0.05, ****p < 0.0001. ns, Nonsignificant.

As Scaf downregulation affects the rate of NB proliferation under normal nutritional conditions, we analyzed whether Scaf overexpression in SPG could enhance neurogenesis in animals subjected to NR. We found that in NR conditions, expression of Scaf in SPG resulted in a 13.78% increase in the size of the adult brain in comparison to control NR animals (Fig. 9*A–C*). Furthermore, the larval central brain NB mitotic index was also increased on Scaf overexpression in animals under NR (Fig. 9*D*). For the BBB, Scaf overexpression did not prevent the reduction in the growth of the SPG or PG layers during NR (Fig. 9*E–J*). Importantly, we did not observe a difference in brain growth during normal Fed conditions on Scaf overexpression (Fig. 9*K–M*), showing that Scaf overexpression enhances brain growth only under starvation. In the case of the growth of other organs, reexpression of Scaf in the BBB during NR, induced a small increase in the size of adult wings (4.76% of increase compared with control animals; Fig. 9*N–R*).

Together, these results demonstrate that Scaf, expressed by the SPG layer, is required for maintaining the rate of neurogenesis. This highlights a nonautonomous role for Scaf in coordinating neurogenesis and the growth of the BBB. Additionally, Scaf reexpression is able to promote brain growth during nutrient deprivation.

### Drug sensitivity is increased on *scarface* loss in the blood–brain barrier

We found that Scaf modulates the growth of the BBB and has a role in maintaining the rate of NB proliferation in the central brain. However, the loss of *scaf* seemed to have only a mild effect in the development of the CNS. Therefore, we wonder whether the phenotypes observed after *scaf* knockdown during larval development has an impact on the function of the BBB and the CNS of the adult animal.

**Figure 10.**
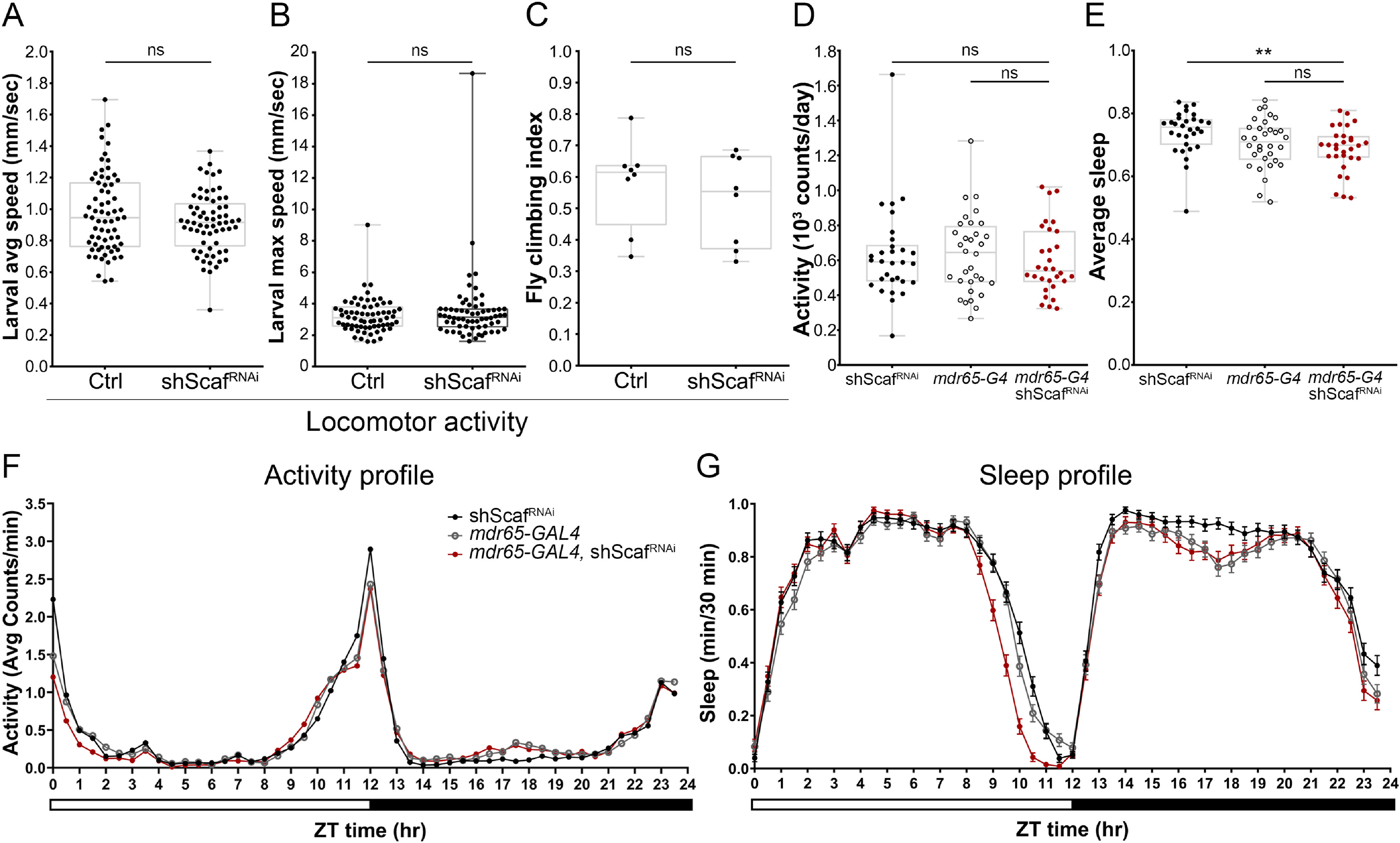
Locomotion activity is not altered on *scaf* knockdown in the blood–brain barrier. ***A–C***, Locomotion assays of *mdr65-GAL4* animals crossed to *w^1118^* (control) and *shScaf^RNAi^*. Graphs showing the larval (***A***) average and (***B***) maximum speed. *n* = 67 and 65 larvae. ***C***, Plot of the adult climbing index using a countercurrent apparatus. *n* = 8 groups of ∼25 adult flies for each genotype. Unpaired Student's *t* test was performed. ***D***, Activity measurements using the DAM2 system. Plot shows the daily number of counts per day of single adult flies of *w^1118^* crossed to *UAS-shScaf^RNAi^* (dark dots, control), *mdr65-GAL4* crossed to *w^1118^* (open dots, control) and *mdr65-GAL4* crossed to *UAS-shScaf^RNAi^* (red dots). *n* = 30, 32, and 30 adult flies, respectively. One-way ANOVA and Dunnett's multiple-comparisons test were performed. ***E***, Sleep assay using the DAM2 system. Graph showing the average sleep fraction per day of single adult flies of the genotypes *w^1118^* crossed to *UAS-shScaf^RNAi^* (dark dots, control), *mdr65-GAL4* crossed to *w^1118^* (open dots, control) and *mdr65-GAL4* crossed to *UAS-shScaf^RNAi^* (red dots). *n* = 30, 32, and 30 adult flies, respectively. One-way ANOVA and Dunnett's multiple comparisons test were performed. **p < 0.01. ***F***, ***G***, Activity (***F***) and sleep profiles (***G***) showing average values in each time point in a 12:12 light:dark cycle. Adult flies of the genotypes: *w^1118^* crossed to *UAS-shScaf^RNAi^* (dark dots, control), *mdr65-GAL4* crossed to *w^1118^* (open dots, control), and *mdr65-GAL4* crossed to *UAS-shScaf^RNAi^* (red dots). *n* = 30, 32, and 30 adult flies respectively. ZT, Zeitgeber time; ns, nonsignificant.

With this goal, we analyzed locomotor activity in larval and adult animals on *scaf* knockdown in SPG. We found no significant differences in the larval crawling speed (average and maximum) of control and *scaf* knockdown animals (Fig. 10*A*,*B*). Similar results were obtained when we assessed climbing activity in adult flies (Fig. 10*C*). As BBB function has been associated with circadian activity rhythm and sleep (Artiushin et al., 2018; Zhang et al., 2018; Cuddapah et al., 2019), we analyzed locomotor activity and sleep in adult flies using DAM2. We found no differences in total activity or activity profile between control and *scaf* knockdown groups (Fig. 10*D*,*F*). Total sleep was not affected in *scaf* knockdown animals either (note that driver-alone sleep did not differ from *scaf* knockdown group in Fig. 10*E*), however, the sleep profile of these animals showed less sleep than both control groups a few hours before the end of the light cycle (Fig. 10*G*).

**Figure 11.**
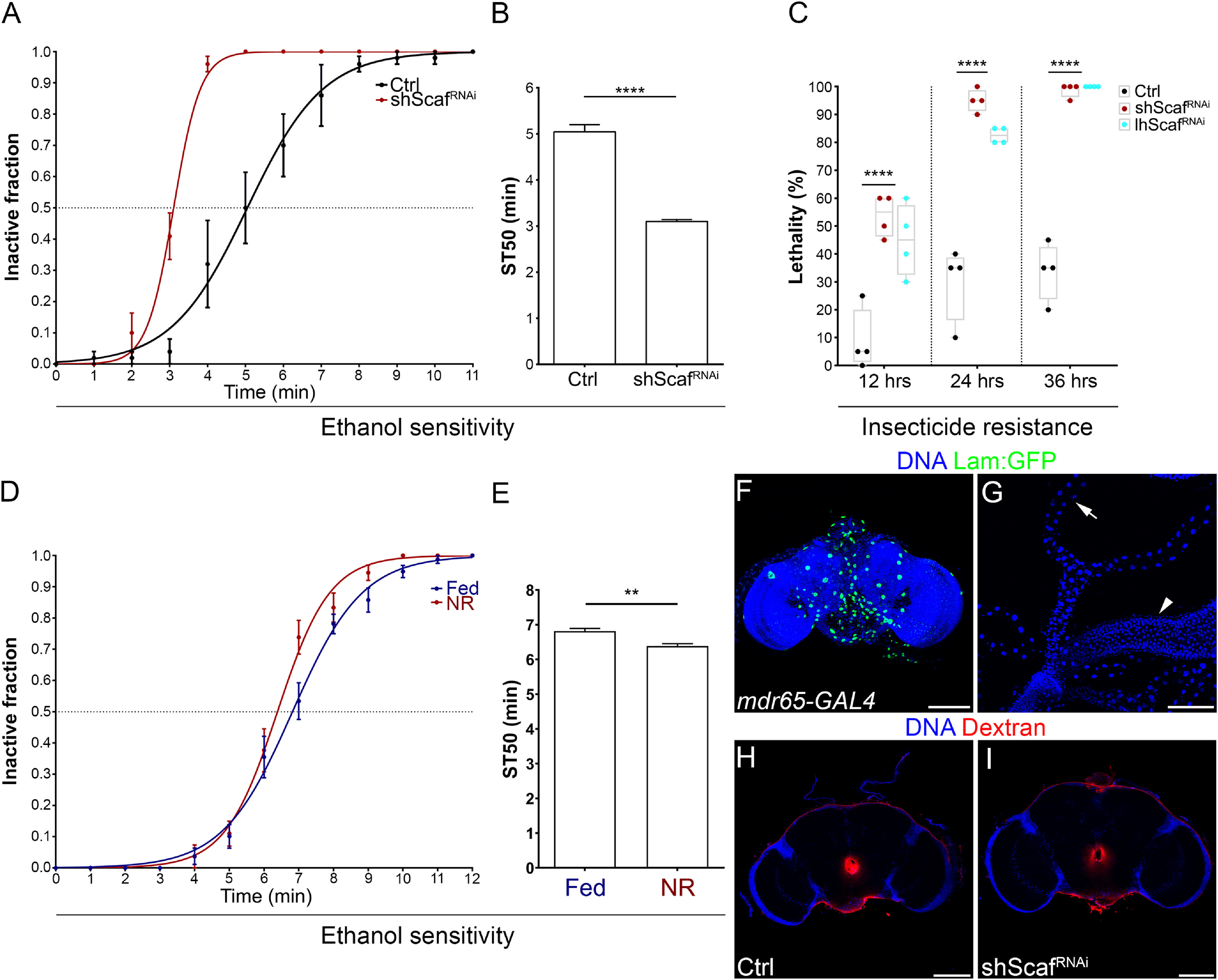
Drug sensitivity is affected by *scaf* knockdown in the blood–brain barrier. ***A***, ***B***, Ethanol sensitivity assay. ***A***, Graph showing the inactive fraction of flies at different time points. Adult flies of *mdr65-GAL4* animals crossed to *w^1118^* (control, dark dots) and *UAS-shScaf^RNAi^* (*scaf* knockdown, red dots). ***B***, Sedation time 50 (ST50) was calculated from ***A***. ***B***, Bar chart showing ST50 for each genotype. *n* = 5 groups of 8–10 adult flies for each genotype. Unpaired Student's *t* test was performed. ***C***, Insecticide resistance assay. Adult flies of *mdr65-GAL4* crossed to *w^1118^* (control, dark dots), *UAS-shScaf^RNAi^* (*scaf* knockdown, red dots) and *UAS-lhScaf^RNAi^* (*scaf* knockdown, cyan dots). Plot shows the lethal fraction after 12, 24, and 36 h of exposure to 0.01% malathion. *n* = 4 groups of 20 adult flies for each genotype. Two-way ANOVA and Dunnett's multiple-comparisons test were done. ***D***, ***E***, Ethanol sensitivity assay of male *w^1118^* flies that grew under Fed or NR conditions. ***D***, Graph showing the inactive fraction of flies at different time points. Fed (blue) and NR (red). ***E***, Sedation time 50 (ST50) was calculated from ***D***. ***E***, Bar chart showing ST50 for Fed and NR conditions. *n* = 8 and 6 groups of 8 adult flies each. Unpaired Student's *t* test was performed. **p < 0.01, ****p < 0.0001. ***F***, ***G***, Expression of the *mdr65-GAL4* driver in adult tissues. (***F***) Images of the adult brain and (***G***) hindgut (arrowhead) and malpighian tubules (arrow). DAPI was used to stain DNA (blue) and *UAS-lam:GFP* (green) for marking nuclei. ***H***, ***I***, Blood–brain barrier permeability assay in *mdr65-GAL4* animals crossed to (***H***) *w^1118^* (control) or (***I***) *UAS-shScaf^RNAi^*. Images show adult brains; DNA was stained with DAPI in blue and 10 kDa dextran-Rhodamine in red. Scale bars: 100 µm.

One of the major roles of the BBB is to control the efflux and influx of molecules across the brain, and it is known that the sensitivity to different drugs is altered in mutant animals with defects in BBB permeability (Bainton et al., 2005; Mayer et al., 2009). The BBB permeability is controlled by preventing paracellular diffusion (by septate junctions) and by the active extrusion of lipophilic molecules into the hemolymph. Hence, we assessed the sensitivity to ethanol and malathion as a manner to test for BBB function defects. Ethanol, although attractive for the flies, produces incoordination and loss of equilibrium in flies (pass out), and its tolerance is regulated in the BBB by the action of A kinase anchoring protein in PG (Parkhurst et al., 2018). Malathion is an organophosphate insecticide that blocks acetylcholinesterase, inducing hyperexcitability as acetylcholine is the main excitatory neurotransmitter in the fly brain. In *Drosophila*, malathion sensitivity is increased by the loss of the ATP-binding cassette transporter *mdr65* (Sun et al., 2017), which is necessary for BBB xenobiotic efflux (Mayer et al., 2009; Hindle et al., 2017). Similarly, in vertebrates malathion can cross and affect the structure of the BBB (Balbuena et al., 2010, 2011). We found that sensitivity to a single exposure of ethanol was significantly enhanced in animals with *scaf* knockdown in SPG (Fig. 11*A*,*B*), and animals exposed to malathion, showed a significant increase in lethality compared with control animals (Fig. 11*C*). Interestingly, adult flies that were subjected to larval NR, in which *scaf* is downregulated in the BBB, also showed an increased sensitivity to ethanol (Fig. 11*D*,*E*). Importantly, the sensitivity to drugs after *scaf* knockdown could not be attributed to an effect over the excretion of drugs because *scaf* RNAi was not expressed in the adult gut or renal (Malpighian) tubules (Fig. 11*F*,*G*, *mdr65-GAL4* expression). Additionally, paracellular permeability of the adult BBB was not affected by *scaf* knockdown (Fig. 11*H*,*I*), suggesting that extrusion of xenobiotic agents may be affected by the loss of *scaf* in the BBB.

These results show that reducing the expression of *scaf* in the BBB, which has an effect over BBB growth, increases the sensitivity of the CNS to exogenous agents, supporting a role of Scaf in the regulation of the function of the BBB.

## Discussion

The adaptive response of animals to periods of famine is essential for species survival. In the CNS, the brain is preferentially protected over other tissues during starvation. Here, we analyze the transcriptional response of *Drosophila melanogaster* NBs and BBB glial cells to NR during larval development using TaDa. Overall, we found differentially expressed genes in NBs, subperineurial and surface glial datasets. We also found that in our starvation model, the major effect of NR was on the growth of the BBB. Thus, we focused on the function of a gene that is highly regulated by NR in the BBB, the SPH *scarface* (*scaf*). We determined that *scaf* is expressed in the SPG, and its expression is modulated by the animal's nutritional state. Tissue-specific knockdown of *scaf* showed that it is required to restrict the growth of the BBB and to maintain a proper rate of NB proliferation. In accordance with this, reintroducing Scaf expression in SPG enhances neurogenesis in animals subjected to nutritional stress. Finally, we showed that *scaf* knockdown in the BBB increases sensitivity to drugs in adult animals, suggesting defects in the control of the influx/efflux of molecules across the BBB.

### A genetic response of the CNS to food scarcity

The maintenance of homeostasis during inanition permits animal survival and developmental progression. Among the different organs, the CNS, which is extremely sensitive to stress conditions, is protected from systemic nutrient reduction. The brain-sparing model is good example of this mechanism of protection during development. In *Drosophila*, neural stem cell proliferation depends on nutrition at early larval stages (Britton and Edgar, 1998; Chell and Brand, 2010; Sousa-Nunes et al., 2011), but it is independent of nutrition through the end of third instar larval stage (after acquiring the minimal viable weight; Cheng et al., 2011). Cheng et al. (2011) said that NBs maintain their proliferation rate by the action of Jelly belly/Anaplastic lymphoma kinase that activates the phosphatidylinositol 3-kinase pathway. However, these pathways do not respond to starvation, nor are they modulated by the nutritional state of the animal (Cheng et al., 2011). This raised the question of whether neural stem cells respond to, or are insensitive to, nutrient restriction. It is plausible that molecular adaptation to nutritional stress occurs in other cell types, such as glial cells, or in other organs.

Our TaDa data showed that there are transcriptional changes in NBs during NR that could help to maintain proliferation and neurogenesis. We postulate that the decrease in the expression of genes associated with ribosome and mitochondrial metabolism could help to maintain the rate of NB proliferation on nutrient restriction. During pupal development, oxidative phosphorylation is required for NBs to reduce growth and exit the cell cycle (Homem et al., 2014; van den Ameele and Brand, 2019), but it is also necessary for larval NBs and NB-derived tumor proliferation (Genovese et al., 2019; van den Ameele and Brand, 2019). Hence, modulating mitochondrial metabolism could be an NB response for maintaining neurogenesis during nutrient restriction. However, the actual contribution of this metabolic regulation to neurogenesis is unknown.

On the other hand, it is well established that other organs contribute to the brain-sparing phenomenon. For instance, on starvation polyploid tissues such as salivary glands and body fat stop growing (Cheng et al., 2011), and body-fat-derived glycogen maintains trehalose levels in the hemolymph (Yamada et al., 2018). Therefore, nutrient stores are mostly available for the CNS to continue growing. The role of neurons and glial cells in this process is less understood. In the case of hypoxia, NB proliferation is protected by the formation of lipid droplets in glial cells (Bailey et al., 2015), supporting a protective role of glial cells. In recent work, the Simon Sprecher group characterized the response of the first instar larval brain to starvation using single-cell sequencing. Although, under these conditions neurogenesis is completely blocked, the group found a transcriptional response in glial cells to promote lipid catabolism (Brunet Avalos et al., 2019). The glia forming the BBB are likely to be the first sensors of nutrient reduction in the hemolymph. The presence of different macromolecule transporters in the BBB (DeSalvo et al., 2014; Volkenhoff et al., 2015; Galagovsky et al., 2018) and their capacity to store neutral lipids (Bailey et al., 2015) supports the role of the BBB as the nutritional gatekeeper of the brain.

In our analysis, we observed that SPG showed an important transcriptional response to nutrient restriction. Among the differentially expressed genes, we found secreted proteins and components of the JNK and JAK/STAT signaling that are well-characterized stress-response pathways. Recently, Prasad and Hens (2018) analyzed the transcriptional response of the adult brain to starvation and sugar absence, finding many secreted proteins, including serine protease homologs and Scaf secreted from a group of neurons (i.e., scarface neurons in the adult brain), which promote feeding behavior. Thus, it is plausible that changes in the expression of secreted proteins, including Scaf, are part of a signature of the brain response to nutrient restriction. Surprisingly, we found that the activity of the JNK and JAK/STAT pathways in the BBB was not affected by nutrient restriction. It would be interesting to evaluate whether during nutrient restriction a decrease in components of these pathways behave as signals from the BBB to other tissues. This is possible considering that Unpaired cytokines and the TNF Eiger from the fat body regulate homeostasis in a systemic manner, which also depends on nutrition (Rajan and Perrimon, 2012; Agrawal et al., 2016).

### Scarface coordinates blood–brain barrier growth with neurogenesis in a cell nonautonomous manner

From our TaDa analysis, we selected Scaf as a good candidate to mediate BBB adaptation to nutrient restriction. Scaf belongs to the SPH family, which lacks amidase activity (Ross et al., 2003; Bonin and Mann, 2004). Scaf was previously described to be a transcriptional target of the JNK pathway, which controls epithelial polarity and morphogenesis during dorsal closure (Rousset et al., 2010; Sorrosal et al., 2010; Kushnir et al., 2017) and thoracic development (Srivastava and Dong, 2015). We showed that Scaf expression in SPG depends on nutrition, and one of its roles is to reduce SPG growth by endocycle/endomitosis as well as PG proliferation. Although, the effect of *scaf* loss is not strong enough to affect animal development to adulthood, sensitivity to drugs that cross the BBB is enhanced, revealing a physiological consequence to this defect in the growth of the BBB. We postulate that loss of *scaf* could affect transcellular diffusion of organic molecules across the BBB or the function of efflux transporters that eliminate xenobiotics from the brain (Mayer et al., 2009; Hindle et al., 2017; Sun et al., 2017).

How Scaf regulates the growth of the BBB is unknown. As Scaf has been shown to be a secreted protein (Rousset et al., 2010; Sorrosal et al., 2010), we propose that Scaf from SPG modulates the activity of growth factors for both SPG and PG. The balance between SPG endocycle and endomitosis is regulated by the Notch pathway (Von Stetina et al., 2018). This mechanism seems to be an autocrine signaling because δ ligand is also necessary in SPG for promoting endocycle over endomitosis (Von Stetina et al., 2018). On the other hand, PG proliferates in response to insulin and FGF signaling (Franzdóttir et al., 2009; Avet-Rochex et al., 2012). Therefore, it is possible that Scaf could affect these signaling pathways to reduce the growth of the BBB. Given that Scaf represses the JNK pathway during embryogenesis, it was postulated that Scaf could act on an unknown extracellular signal, including components of the extracellular matrix (Rousset et al., 2010). Spheroid, another SPH, regulates Toll pathway activation in a bacterial infection model (Patrnogic and Leclerc, 2017), whereas the SPH Masquerade regulates muscle attachment by stabilizing cell–matrix interactions (Murugasu-Oei et al., 1995). We showed that Scaf reaches the entire BBB; therefore, Scaf may play a similar role in modulating signaling pathways in the BBB as other SPHs. Alternatively, Scaf could also have a role intracellularly because it can be found in early, late, and recycling endosomes in neighboring cells (Sorrosal et al., 2010).

Interestingly, Scaf displays a cell nonautonomous effect over NB proliferation. We did not detect Scaf in NBs, therefore, this effect is indirect. One possibility is that reducing BBB growth could have an impact on nutrient availability inside the brain. This could explain the fact that Scaf overexpression during nutrient restriction enhances brain growth. Similarly, a reduction in BBB growth, mediated by Scaf, could increase the secretion of growth factors such as insulin-like peptides and activin that control NB proliferation (Zhu et al., 2008; Chell and Brand, 2010; Sousa-Nunes et al., 2011). Additionally, its overexpression in the BBB did not rescue the proliferation effect observed in the mutants, suggesting that other glial cells, such as cortex glia, could be expressing Scaf and contributing to the neurogenic niche.

We believe that the decrease in Scaf levels is part of a highly complex mechanism to maintain growth in the BBB during NR. Knocking down *scaf* in the BBB does not completely recapitulate the phenotype observed during starvation, suggesting that other pathways are affected. Moreover, reexpression of Scaf during NR does not rescue BBB growth. We propose that during normal development, Scaf responds to nutrient availability and acts as a negative feedback over BBB growth, contributing to the fine-tuning of the balance between BBB growth and neurogenesis in the CNS. During larval starvation, this balance is lost, and neurogenesis is prioritized at the expense of the growth of the BBB. Future experimental evidence will be needed to unveil the molecular mechanisms by which Scaf supports this balance of growth during the development of the nervous system.

## References

[B1] Agrawal N, Delanoue R, Mauri A, Basco D, Pasco M, Thorens B, Léopold P (2016) The *Drosophila* TNF Eiger is an adipokine that acts on insulin-producing cells to mediate nutrient response. Cell Metab 23:675–684. 10.1016/j.cmet.2016.03.003 27076079

[B2] Albertson R, Chabu C, Sheehan A, Doe CQ (2004) Scribble protein domain mapping reveals a multistep localization mechanism and domains necessary for establishing cortical polarity. J Cell Sci 117:6061–6070. 15536119 10.1242/jcs.01525

[B3] Artiushin G, Zhang SL, Tricoire H, Sehgal A (2018) Endocytosis at the *Drosophila* blood–brain barrier as a function for sleep. Elife 7:e43326. 10.7554/eLife.43326 30475209 PMC6255390

[B4] Aughey GN, Southall TD (2016) Dam it's good! DamID profiling of protein-DNA interactions. Wiley Interdiscip Rev Dev Biol 5:25–37. 10.1002/wdev.205 26383089 PMC4737221

[B5] Avet-Rochex A, Kaul AK, Gatt AP, McNeill H, Bateman JM (2012) Concerted control of gliogenesis by InR/TOR and FGF signalling in the *Drosophila* post-embryonic brain. Development 139:2763–2772. 10.1242/dev.074179 22745312 PMC3392704

[B6] Awasaki T, Lai S-L, Ito K, Lee T (2008) Organization and postembryonic development of glial cells in the adult central brain of *Drosophila*. J Neurosci 28:13742–13753. 10.1523/JNEUROSCI.4844-08.2008 19091965 PMC6671902

[B7] Bach EA, Ekas LA, Ayala-Camargo A, Flaherty MS, Lee H, Perrimon N, Baeg G-H (2007) GFP reporters detect the activation of the Drosophila JAK/STAT pathway in vivo. Gene Expr Patterns 7:323–331. 10.1016/j.modgep.2006.08.003 17008134

[B8] Bailey AP, Koster G, Guillermier C, Hirst EMA, MacRae JI, Lechene CP, Postle AD, Gould AP (2015) Antioxidant role for lipid droplets in a stem cell niche of *Drosophila*. Cell 163:340–353. 10.1016/j.cell.2015.09.020 26451484 PMC4601084

[B9] Bainton RJ, Tsai LTY, Schwabe T, DeSalvo M, Gaul U, Heberlein U (2005) moody encodes two GPCRs that regulate cocaine behaviors and blood–brain barrier permeability in *Drosophila*. Cell 123:145–156. 10.1016/j.cell.2005.07.029 16213219

[B10] Balbuena P, Li W, Ehrich M (2011) Assessments of tight junction proteins occludin, claudin 5 and scaffold proteins ZO1 and ZO2 in endothelial cells of the rat blood–brain barrier: cellular responses to neurotoxicants malathion and lead acetate. Neurotoxicology 32:58–67. 10.1016/j.neuro.2010.10.004 20970449

[B11] Balbuena P, Li W, Magnin-Bissel G, Meldrum JB, Ehrich M (2010) Comparison of two blood–brain barrier in vitro systems: cytotoxicity and transfer assessments of malathion/oxon and lead acetate. Toxicol Sci 114:260–271. 10.1093/toxsci/kfq001 20064834

[B12] Baumgartner S, Littleton JT, Broadie K, Bhat MA, Harbecke R, Lengyel JA, Chiquet-Ehrismann R, Prokop A, Bellen HJ (1996) A *Drosophila* neurexin is required for septate junction and blood-nerve barrier formation and function. Cell 87:1059–1068. 10.1016/S0092-8674(00)81800-0 8978610

[B13] Benmimoun B, Papastefanaki F, Périchon B, Segklia K, Roby N, Miriagou V, Schmitt C, Dramsi S, Matsas R, Spéder P (2020) An original model of brain infection identifies the hijacking of host lipoprotein import as a bacterial strategy for blood–brain barrier crossing. Nat Commun 11:6106. 10.1038/s41467-020-19826-2 33257684 PMC7704634

[B14] Bonin CP, Mann RS (2004) A piggyBac transposon gene trap for the analysis of gene expression and function in Drosophila. Genetics 167:1801–1811. 10.1534/genetics.104.027557 15342518 PMC1470976

[B15] Brand AH, Perrimon N (1993) Targeted gene expression as a means of altering cell fates and generating dominant phenotypes. Development 118:401–415. 10.1242/dev.118.2.401 8223268

[B16] Britton JS, Edgar BA (1998) Environmental control of the cell cycle in *Drosophila*: nutrition activates mitotic and endoreplicative cells by distinct mechanisms. Development 125:2149–2158. 10.1242/dev.125.11.2149 9570778

[B17] Brooks DS, Vishal K, Kawakami J, Bouyain S, Geisbrecht ER (2016) Optimization of wrMTrck to monitor *Drosophila* larval locomotor activity. J Insect Physiol 93–94:11–17. 10.1016/j.jinsphys.2016.07.007 PMC5722213 27430166

[B18] Brunet Avalos C, Maier GL, Bruggmann R, Sprecher SG (2019) Single cell transcriptome atlas of the *Drosophila* larval brain. Elife 8:e50354. 10.7554/eLife.50354 31746739 PMC6894929

[B19] Carlson SD, Juang J-L, Hilgers SL, Garment MB (2000) Blood barriers of the insect. Annu Rev Entomol 45:151–174. 10.1146/annurev.ento.45.1.151 10761574

[B20] Chatterjee N, Bohmann D (2012) A versatile φC31 based reporter system for measuring AP-1 and NRF2 signaling in *Drosophila* and in tissue culture. PLoS One 7:e34063. 10.1371/journal.pone.0034063 22509270 PMC3324472

[B21] Chell JM, Brand AH (2010) Nutrition-responsive glia control exit of neural stem cells from quiescence. Cell 143:1161–1173. 10.1016/j.cell.2010.12.007 21183078 PMC3087489

[B22] Cheng LY, Bailey AP, Leevers SJ, Ragan TJ, Driscoll PC, Gould AP (2011) Anaplastic lymphoma kinase spares organ growth during nutrient restriction in *Drosophila*. Cell 146:435–447. 10.1016/j.cell.2011.06.040 21816278

[B23] Chiu JC, Low KH, Pike DH, Yildirim E, Edery I (2010) Assaying locomotor activity to study circadian rhythms and sleep parameters in *Drosophila*. J Vis Exp 9 28:2157. 20972399 10.3791/2157 PMC3229366

[B24] Cichewicz K, Hirsh J (2018) ShinyR-DAM: a program analyzing Drosophila activity, sleep and circadian rhythms. Commun Biol 1:25. 10.1038/s42003-018-0031-9 29911688 PMC6002956

[B25] Cohen E, Baerts W, Van Bel F (2015) Brain-sparing in intrauterine growth restriction: considerations for the neonatologist. Neonatology 108:269–276. 10.1159/000438451 26330337

[B26] Contreras EG, Sierralta J, Glavic A (2018) p53 is required for brain growth but is dispensable for resistance to nutrient restriction during *Drosophila* larval development. PLoS One 13:e0194344. 10.1371/journal.pone.0194344 29621246 PMC5886404

[B27] Cuddapah VA, Zhang SL, Sehgal A (2019) Regulation of the blood–brain barrier by circadian rhythms and sleep. Trends Neurosci 42:500–510. 10.1016/j.tins.2019.05.001 31253251 PMC6602072

[B28] DeSalvo MK, Hindle SJ, Rusan ZM, Orng S, Eddison M, Halliwill K, Bainton RJ (2014) The *Drosophila* surface glia transcriptome: evolutionary conserved blood–brain barrier processes. Front Neurosci 8:1–22. 25426014 10.3389/fnins.2014.00346 PMC4224204

[B29] Franzdóttir SR, Engelen D, Yuva-Aydemir Y, Schmidt I, Aho A, Klämbt C (2009) Switch in FGF signalling initiates glial differentiation in the *Drosophila* eye. Nature 460:758–761. 10.1038/nature08167 19597479

[B30] Frawley LE, Orr-Weaver TL (2015) Primer polyploidy. Curr Biol 25:353–358. 10.1016/j.cub.2015.03.037 25942544

[B31] Galagovsky D, Depetris-Chauvin A, Manière G, Geillon F, Berthelot-Grosjean M, Noirot E, Alves G, Grosjean Y (2018) Sobremesa L-type amino acid transporter expressed in glia is essential for proper timing of development and brain growth. Cell Rep 24:3156–3166.e4. 10.1016/j.celrep.2018.08.067 30231999 PMC6167638

[B32] Genovese S, Clément R, Gaultier C, Besse F, Narbonne-Reveau K, Daian F, Foppolo S, Luis NM, Maurange C (2019) Coopted temporal patterning governs cellular hierarchy, heterogeneity and metabolism in Drosophila neuroblast tumors. Elife 8:e50375. 10.7554/eLife.50375 31566561 PMC6791719

[B33] González-Itier S, Contreras E, Larraín J, Glavic Á, Faunes F (2018) A role for Lin-28 in growth and metamorphosis in *Drosophila melanogaster*. Mech Dev 154:107–115. 10.1016/j.mod.2018.06.002 29908237

[B34] Gürsoy-özdemir Y, Tas YC (2017) Anatomy and Physiology of the Blood–brain Barrier. Nanotechnol Methods Neurol Dis Brain Tumors Drug Deliv across Blood–brain Barrier 38:1–13.

[B35] Haddad-Tóvolli R, Dragano NRV, Ramalho AFS, Velloso LA (2017) Development and function of the blood–brain barrier in the context of metabolic control. Front Neurosci 11:1–12. 28484368 10.3389/fnins.2017.00224 PMC5399017

[B36] Handke B, Poernbacher I, Goetze S, Ahrens CH, Omasits U, Marty F, Simigdala N, Meyer I, Wollscheid B, Brunner E, Hafen E, Lehner CF (2013) The hemolymph proteome of fed and starved *Drosophila* larvae. PLoS One 8:e67208–10. 10.1371/journal.pone.0067208 23840627 PMC3688620

[B37] Hindle SJ, Bainton RJ (2014) Barrier mechanisms in the *Drosophila* blood–brain barrier. Front Neurosci 8:1–12. 25565944 10.3389/fnins.2014.00414 PMC4267209

[B38] Hindle SJ, Munji RN, Dolghih E, Gaskins G, Orng S, Ishimoto H, Soung A, DeSalvo M, Kitamoto T, Keiser MJ, Jacobson MP, Daneman R, Bainton RJ (2017) Evolutionarily conserved roles for blood–brain barrier xenobiotic transporters in endogenous steroid partitioning and behavior. Cell Rep 21:1304–1316. 10.1016/j.celrep.2017.10.026 29091768 PMC5774027

[B39] Homem CCF, Steinmann V, Burkard TR, Jais A, Esterbauer H, Knoblich JA (2014) Ecdysone and mediator change energy metabolism to terminate proliferation in *Drosophila* neural stem cells. Cell 158:874–888. 10.1016/j.cell.2014.06.024 25126791

[B40] Huang DW, Sherman BT, RA L (2009a) Bioinformatics enrichment tools: paths toward the comprehensive functional analysis of large gene lists. Nucleic Acids Res 37:1–13. 19033363 10.1093/nar/gkn923 PMC2615629

[B41] Huang DW, Sherman BT, Lempicki RA (2009b) Systematic and integrative analysis of large gene lists using DAVID bioinformatics resources. Nat Protoc 4:44–57. 10.1038/nprot.2008.211 19131956

[B42] Inagaki HK, Kamikouchi A, Ito K (2010) Methods for quantifying simple gravity sensing in *Drosophila melanogaster*. Nat Protoc 5:20–25. 10.1038/nprot.2009.196 20010724

[B43] Jenett A, Rubin GM, Ngo T-TB, Shepherd D, Murphy C, Dionne H, Pfeiffer BD, Cavallaro A, Hall D, Jeter J, Iyer N, Fetter D, Hausenfluck JH, Peng H, Trautman ET, Svirskas RR, Myers EW, Iwinski ZR, Aso Y, DePasquale GM, et al. (2012) A GAL4-driver line resource for *Drosophila* neurobiology. Cell Rep 2:991–1001. 10.1016/j.celrep.2012.09.011 23063364 PMC3515021

[B44] Kanai MI, Kim MJ, Akiyama T, Takemura M, Wharton K, O'Connor MB, Nakato H (2018) Regulation of neuroblast proliferation by surface glia in the *Drosophila* larval brain. Sci Rep 8:15. 10.1038/s41598-018-22028-y 29487331 PMC5829083

[B45] Koh YH, Popova E, Thomas U, Griffith LC, Budnik V (1999) Regulation of DLG localization at synapses by CaMKII-dependent phosphorylation. Cell 98:353–363. 10.1016/S0092-8674(00)81964-9 10458610 PMC4656018

[B46] Kushnir T, Mezuman S, Bar-Cohen S, Lange R, Paroush Z, Helman A (2017) Novel interplay between JNK and Egfr signaling in *Drosophila* dorsal closure. PLoS Genet 13:e1006860–18. 10.1371/journal.pgen.1006860 28628612 PMC5495517

[B47] Lanet E, Gould AP, Maurange C (2013) Protection of neuronal diversity at the expense of neuronal numbers during nutrient restriction in the *Drosophila* visual system. Cell Rep 3:587–594. 10.1016/j.celrep.2013.02.006 23478023 PMC3617362

[B48] Lanet E, Maurange C (2014) Building a brain under nutritional restriction: insights on sparing and plasticity from *Drosophila* studies. Front Physiol 5:117. 24723892 10.3389/fphys.2014.00117 PMC3972452

[B49] Layalle S, Arquier N, Léopold P (2008) The TOR pathway couples nutrition and developmental timing in *Drosophila*. Dev Cell 4:568–577. 10.1016/j.devcel.2008.08.003 18854141

[B50] Li D, Liu Y, Pei C, Zhang P, Pan L, Xiao J, Meng S, Yuan Z, Bi X (2017) miR-285– Yki/Mask double-negative feedback loop mediates blood–brain barrier integrity in *Drosophila*. Proc Natl Acad Sci U S A 114:E2365–E2374. 10.1073/pnas.1613233114 28265104 PMC5373330

[B51] Marshall OJ, Brand AH (2015) Damidseq-pipeline: an automated pipeline for processing DamID sequencing datasets. Bioinformatics 31:3371–3373. 10.1093/bioinformatics/btv386 26112292 PMC4595905

[B52] Marshall OJ, Southall TD, Cheetham SW, Brand AH (2016) Cell-type-specific profiling of protein – DNA interactions without cell isolation using targeted DamID with next-generation sequencing. Nat Protoc 11:1586–1598. 10.1038/nprot.2016.084 27490632 PMC7032955

[B53] Mayer F, Mayer N, Chinn L, Pinsonneault RL, Kroetz D, Bainton RJ (2009) Evolutionary conservation of vertebrate blood–brain barrier chemoprotective mechanisms in *Drosophila*. J Neurosci 29:3538–3550. 10.1523/JNEUROSCI.5564-08.2009 19295159 PMC3040577

[B54] Morin X, Daneman R, Zavortink M, Chia W (2001) A protein trap strategy to detect GFP-tagged proteins expressed from their endogenous loci in *Drosophila*. Proc Natl Acad Sci U S A 98:15050–15055. 10.1073/pnas.261408198 11742088 PMC64981

[B55] Murugasu-Oei B, Rodrigues V, Yang X, Chia W (1995) Masquerade: a novel secreted serine protease-like molecule is required somatic muscle attachment in the *Drosophila* embryo. Genes Dev 9:139–154. 7851790 10.1101/gad.9.2.139

[B56] O'Brown NM, Pfau SJ, Gu C (2018) Bridging barriers: a comparative look at the blood–brain barrier across organisms. Genes Dev 32:466–478. 10.1101/gad.309823.117 29692355 PMC5959231

[B57] Øvrebø JI, Edgar BA (2018) Polyploidy in tissue homeostasis and regeneration. Development 145:dev156034. 10.1242/dev.156034 30021843 PMC10682953

[B58] Parkhurst SJ, Adhikari P, Navarrete JS, Legendre A, Manansala M, Wolf FW (2018) Perineurial barrier glia physically respond to alcohol in an Akap200-dependent manner to promote tolerance. Cell Rep 22:1647–1656. 10.1016/j.celrep.2018.01.049 29444420 PMC5831198

[B59] Patrnogic J, Leclerc V (2017) The serine protease homolog spheroide is involved in sensing of pathogenic Gram-positive bacteria. PLoS One 12:e0188339–13. 10.1371/journal.pone.0188339 29211760 PMC5718610

[B60] Pereanu W, Shy D, Hartenstein V (2005) Morphogenesis and proliferation of the larval brain glia in Drosophila. Dev Biol 283:191–203. 10.1016/j.ydbio.2005.04.024 15907832

[B61] Prasad N, Hens K (2018) Sugar promotes feeding in flies via the serine protease homolog scarface. Cell Rep 24:3194–3206.e4. 10.1016/j.celrep.2018.08.059 30232002 PMC6167639

[B62] Rajan A, Perrimon N (2012) *Drosophila* cytokine unpaired 2 regulates physiological homeostasis by remotely controlling insulin secretion. Cell 151:123–137. 10.1016/j.cell.2012.08.019 23021220 PMC3475207

[B63] Robinson JT, Thorvaldsdóttir H, Winckler W, Guttman M, Lander ES, Getz G, Mesirov JP (2011) Integrative genomics viewer. Nat Biotechnol 29:24–26. 10.1038/nbt.1754 21221095 PMC3346182

[B64] Ross J, Jiang H, Kanost MR, Wang Y (2003) Serine proteases and their homologs in the *Drosophila melanogaster* genome: an initial analysis of sequence conservation and phylogenetic relationships. Gene 304:117–131. 10.1016/s0378-1119(02)01187-3 12568721

[B65] Rousset R, Bono-Lauriol S, Gettings M, Suzanne M, Spéder P, Noselli S (2010) The *Drosophila* serine protease homologue Scarface regulates JNK signalling in a negative-feedback loop during epithelial morphogenesis. Development 137:2177–2186. 10.1242/dev.050781 20530545

[B66] Sandhu S, Kollah AP, Lewellyn L, Chan RF, Grotewiel M (2015) An inexpensive, scalable behavioral assay for measuring ethanol sedation sensitivity and rapid tolerance in *Drosophila*. J Vis Exp 4 15:52676. 10.3791/52676 PMC4423423 25939022

[B67] Schirmeier S, Klämbt C (2015) The *Drosophila* blood–brain barrier as interface between neurons and hemolymph. Mech Dev 138:50–55. 10.1016/j.mod.2015.06.002 26103549

[B68] Schwabe T, Bainton RJ, Fetter RD, Heberlein U, Gaul U (2005) GPCR signaling is required for blood–brain barrier formation in *Drosophila*. Cell 123:133–144. 10.1016/j.cell.2005.08.037 16213218

[B69] Sorrosal G, Pérez L, Herranz H, Milán M (2010) Scarface, a secreted serine protease-like protein, regulates polarized localization of laminin A at the basement membrane of the *Drosophila* embryo. EMBO Rep 11:373–379. 10.1038/embor.2010.43 20379222 PMC2868543

[B70] Sousa-Nunes R, Yee LL, Gould AP (2011) Fat cells reactivate quiescent neuroblasts via TOR and glial insulin relays in *Drosophila*. Nature 471:508–513. 10.1038/nature09867 21346761 PMC3146047

[B71] Southall TD, Gold KS, Egger B, Davidson CM, Caygill EE, Marshall OJ, Brand AH (2013) Cell-type-specific profiling of gene expression and chromatin binding without cell isolation: assaying RNA pol II occupancy in neural stem cells. Dev Cell 26:101–112. 10.1016/j.devcel.2013.05.020 23792147 PMC3714590

[B72] Spéder P, Brand AH (2014) Gap junction proteins in the blood–brain barrier control nutrient-dependent reactivation of *Drosophila* neural stem cells. Dev Cell 30:309–321. 10.1016/j.devcel.2014.05.021 25065772 PMC4139190

[B73] Srivastava A, Dong Q (2015) Regulation of a serine protease homolog by the JNK pathway during thoracic development of *Drosophila melanogaster*. FEBS Open Bio 5:117–123. 10.1016/j.fob.2015.01.008 25737837 PMC4338370

[B74] Stork T, Engelen D, Krudewig A, Silies M, Bainton RJ, Klambt C (2008) Organization and function of the blood brain barrier in *Drosophila*. J Neurosci 28:587–597. 10.1523/JNEUROSCI.4367-07.2008 18199760 PMC6670337

[B75] Sun H, Buchon N, Scott JG (2017) Mdr65 decreases toxicity of multiple insecticides in Drosophila melanogaster. Insect Biochem Mol Biol 89:11–16. 10.1016/j.ibmb.2017.08.002 28803989

[B76] Szklarczyk D, Gable AL, Lyon D, Junge A, Wyder S, Huerta-Cepas J, Simonovic M, Doncheva NT, Morris JH, Bork P, Jensen LJ, Von Mering C (2019) STRING v11: protein-protein association networks with increased coverage, supporting functional discovery in genome-wide experimental datasets. Nucleic Acids Res 47:D607–D613. 10.1093/nar/gky1131 30476243 PMC6323986

[B77] Tapadia MG, Lakhotia SC (2005) Expression of mdr49 and mdr65 multidrug resistance genes in larval tissues of Drosophila melanogaster under normal and stress conditions. Cell Stress Chaperones 10:7–11. 10.1379/csc-67r.1 15832942 PMC1074574

[B78] Uhlirova M, Bohmann D (2006) JNK- and Fos-regulated Mmp1 expression cooperates with Ras to induce invasive tumors in Drosophila. EMBO J 25:5294–5304. 10.1038/sj.emboj.7601401 17082773 PMC1636619

[B79] Unhavaithaya Y, Orr-Weaver TL (2012) Polyploidization of glia in neural development links tissue growth to blood–brain barrier integrity. Genes Dev 26:31–36. 10.1101/gad.177436.111 22215808 PMC3258963

[B80] van den Ameele J, Brand AH (2019) Neural stem cell temporal patterning and brain tumour growth rely on oxidative phosphorylation. Elife 8:e47887. 10.7554/eLife.47887 31513013 PMC6763261

[B81] van den Ameele J, Krautz R, Brand AH (2019) TaDa! Analysing cell type-specific chromatin in vivo with Targeted DamID. Curr Opin Neurobiol 56:160–166. 10.1016/j.conb.2019.01.021 30844670

[B82] Venken KJT, Schulze KL, Haelterman NA, Pan H, He Y, Evans-Holm M, Carlson JW, Levis RW, Spradling AC, Hoskins RA, Bellen HJ (2011) MiMIC: a highly versatile transposon insertion resource for engineering *Drosophila melanogaster* genes. Nat Methods 8:737–743. 10.1038/nmeth.1662 21985007 PMC3191940

[B83] Volkenhoff A, Weiler A, Letzel M, Stehling M, Klämbt C, Schirmeier S (2015) Glial glycolysis is essential for neuronal survival in *Drosophila*. Cell Metab 22:437–447. 10.1016/j.cmet.2015.07.006 26235423

[B84] Von Stetina JR, Frawley LE, Unhavaithaya Y, Orr-Weaver TL (2018) Variant cell cycles regulated by Notch signaling control cell size and ensure a functional blood–brain barrier. Development 145:dev157115. 10.1242/dev.157115 29440220 PMC5818001

[B85] Wu JS, Luo L (2006) A protocol for dissecting *Drosophila melanogaster* brains for live imaging or immunostaining. Nat Protoc 1:2110–2115. 10.1038/nprot.2006.336 17487202

[B86] Yamada T, Habara O, Kubo H, Nishimura T (2018) Fat body glycogen serves as a metabolic safeguard for the maintenance of sugar levels in *Drosophila*. Development 145:dev158865. 10.1242/dev.165910 29467247

[B87] Yildirim K, Petri J, Kottmeier R, Klämbt C (2019) *Drosophila* glia: few cell types and many conserved functions. Glia 67:5–26. 10.1002/glia.23459 30443934

[B88] Zhang SL, Yue Z, Arnold DM, Artiushin G, Sehgal A (2018) A circadian clock in the blood–brain barrier regulates xenobiotic efflux. Cell 173:130–139.e10. 10.1016/j.cell.2018.02.017 29526461 PMC5866247

[B89] Zhao Z, Nelson AR, Betsholtz C, Zlokovic BV (2015) Establishment and dysfunction of the blood–brain barrier. Cell 163:1064–1078. 10.1016/j.cell.2015.10.067 26590417 PMC4655822

[B90] Zhu CC, Boone JQ, Jensen PA, Hanna S, Podemski L, Locke J, Doe CQ, O'Connor MB (2008) *Drosophila* Activin- and the Activin-like product Dawdle function redundantly to regulate proliferation in the larval brain. Development 135:513–521. 10.1242/dev.010876 18171686

