# Supplementary figures and images for: The Serine Protease Homolog, Scarface, Is Sensitive to Nutrient Availability and Modulates the Development of the Drosophila Blood–Brain Barrier
Source: J Neurosci. 2021 Jul 28;41(30):6430–48. doi: 10.1523/JNEUROSCI.0452-20.2021 (PMC8318086; doi:10.1523/JNEUROSCI.0452-20.2021)

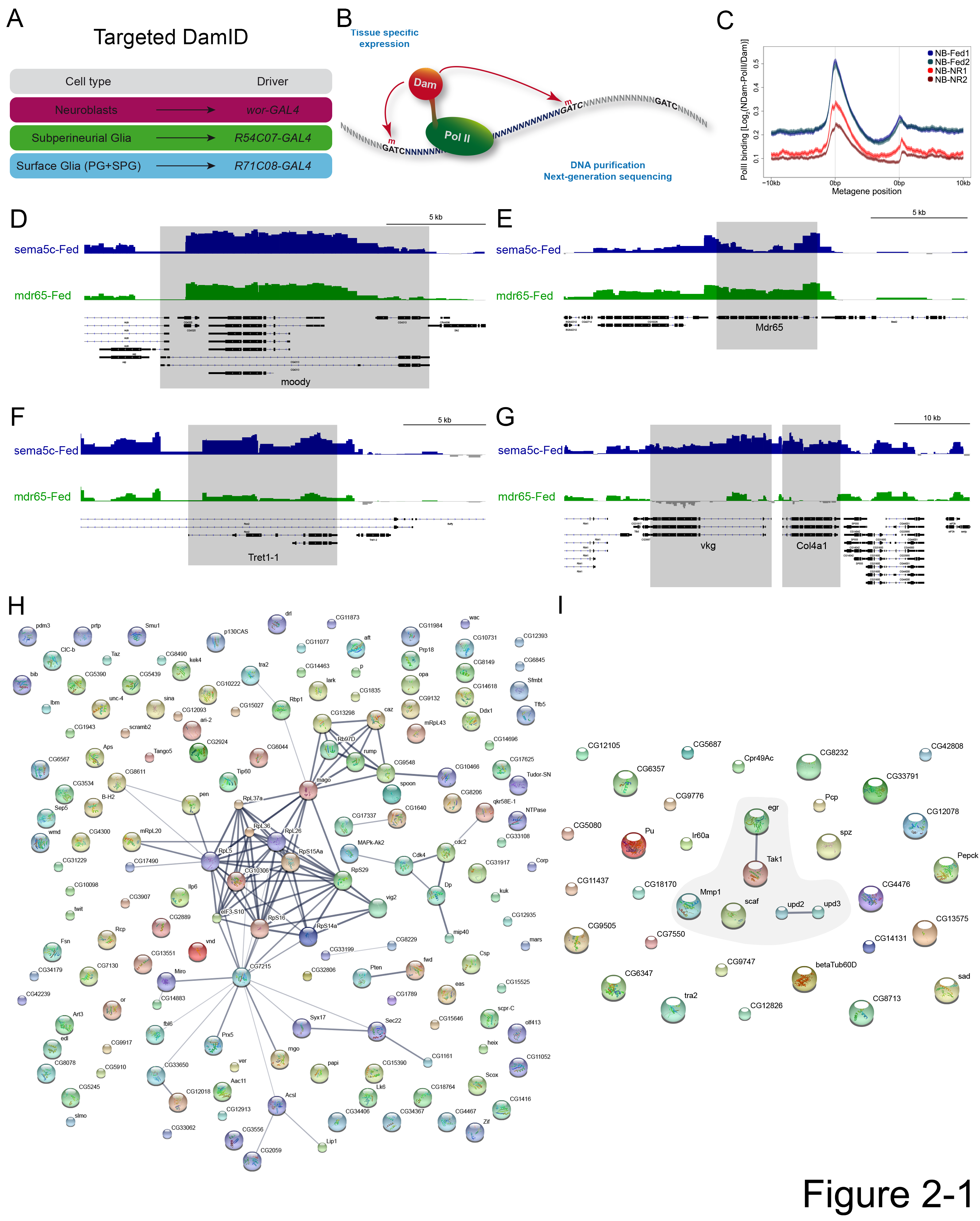

Supplement: Figure 2-1 — A, Drivers used for TaDa in different cell populations. B, Scheme of the TaDa mechanism showing the binding of the NDam-PolII fusion protein to the DNA and the methylation in GATC sequence. C, Meta-analysis of the NDam-PolII binding using the driver wor-GAL4 [neural stem cells (NSC), neuroblast]. Graph shows NDam-PolII in Fed and NR replicates. D–G, Genome view of the (D) moody, (E) mdr65, (F) Tret1-1, and (G) vkg and Col4a1 loci, showing the binding of PolII using sema5c-GAL4 (blue track) and mdr65-GAL4 (green track) drivers under normal Fed conditions. Note that the binding of PolII across moody and mdr65, markers of subperineurial glia, is similar in both drivers; however, the perineurial markers Tret1-1, vkg and Col4g1 are only bound by PolII in sema5c-GAL4 dataset. H, I, Diagram showing a String association network of genes downregulated in (H) NBs and (I) subperineurial glia during NR. Genes associated to the JNK pathway are shown surrounded by a gray background in I. Download Figure 2-1, TIF file. [file ns-JN-RM-0452-20-s01.tif]

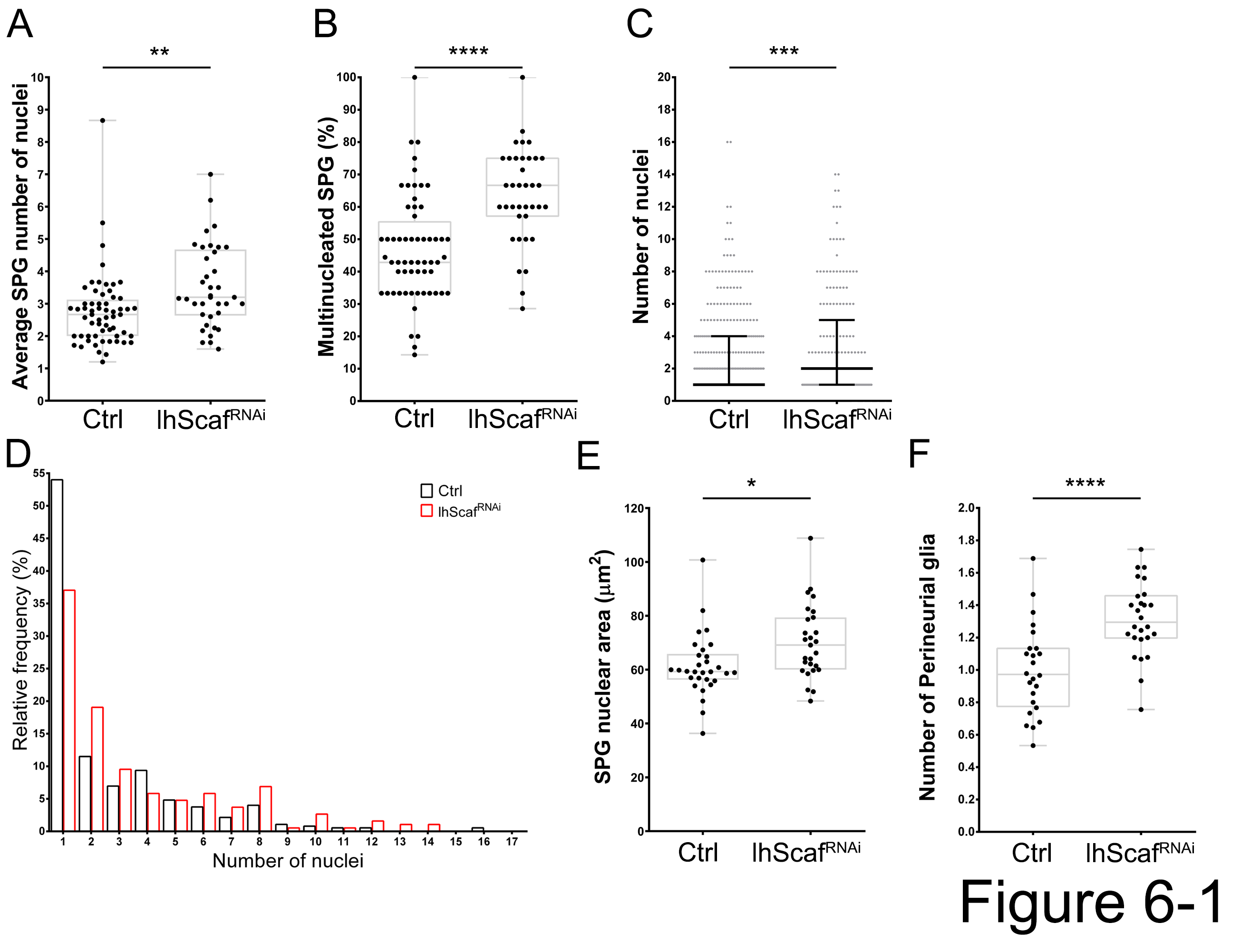

Supplement: Figure 6-1 — A–D, Analysis of larval SPG endomitosis of mdr65-GAL4, UAS-DlgA::GFP animals crossed to w1118 (control) and UAS-lhScafRNAi (scaf knockdown). A, Graph showing the average number of SPG nuclei per brain lobe. n = 60 and 37 brain lobes. Unpaired Student's t test was performed. B, Plot showing the percentage of multinucleated SPG (2 or more nuclei) per brain lobe. n = 60 and 37 brain lobes. Mann–Whitney test was done. C, Plot showing the number of nuclei in each SPG analyzed, median (black line) and interquartile range are shown. Mann–Whitney test was done,. n = 374 and 189 SPG for control (Ctrl) and lhScafRNAi, respectively. D, Histogram depicting the relative distribution (percentage) of SPG according to the number of nuclei. E, Graph showing the average size of the SPG nucleus of larval brains of mdr-GAL4, UAS-DlgA::GFP animals crossed to w1118 (control) and UAS-lhScafRNAi. n = 30 and 27 brain lobes, respectively. Unpaired Student's t test was used. F, Plot showing the distribution of the number of PG per brain lobe. mdr65-GAL4, UAS-GFP.nls were crossed to w1118 (control) and UAS-lhScafRNAi and stained for the glial marker Repo. Repo-positive/GFP-negative nuclei were scored as PG. n = 24 and 26 brain lobes. Unpaired Student's t test was used. *p < 0.05, **p < 0.01, ***p < 0.001, ****p < 0.0001. Download Figure 6-1, TIF file. [file ns-JN-RM-0452-20-s03.tif]
